# Scalalactams A–D, Scalarane Sesterterpenes with a γ-Lactam Moiety from a Korean *Spongia* Sp. Marine Sponge

**DOI:** 10.3390/molecules23123187

**Published:** 2018-12-03

**Authors:** Inho Yang, Jusung Lee, Jihye Lee, Dongyup Hahn, Jungwook Chin, Dong Hwan Won, Jaeyoung Ko, Hyukjae Choi, Ahreum Hong, Sang-Jip Nam, Heonjoong Kang

**Affiliations:** 1 Department of Convergence Study on the Ocean Science and Technology, Korea Maritime and Ocean University, Busan 49112, Korea; ihyang@kmou.ac.kr; 2 The Center for Marine Natural Products and Drug Discovery, School of Earth and Environmental Science, Seoul National University, NS-80, Seoul 08826, Korea; leejusung@snu.ac.kr (J.L.); jl3414@gmail.com (J.L.); dongfal2@gmail.com (D.H.W.); 3 School of Food Science and Biotechnology, Kyungpook National University, Daegu 41566, Korea; dohahn@knu.ac.kr; 4 Institute of Agricultural Science & Technology, Kyungpook National University, Daegu 41566, Korea; 5 New Drug Development Center, Daegu-Gyeongbuk Medicinal Innovation Foundation, Daegu 41061, Korea; jwchin@dgmif.re.kr; 6 Basic Research & Innovation Division, Amorepacific R&D Unit, Yongin 17074, Korea; jaeyoungko@amorepacific.com; 7 College of Pharmacy, Yeungnam University, Gyeongsan 38541, Korea; h5choi@yu.ac.kr; 8 Graduate School of Industrial Pharmaceutical Sciences, Ewha Womans University, Seoul 03760, Korea; lyzenne@gmail.com; 9 Department of Chemistry and Nanoscience, Ewha Womans University, Seoul 03760, Korea; 10 Research Institute of Oceanography, Seoul National University, NS-80, Seoul 08826, Korea

**Keywords:** scalarane sesterterpenes, scalalactams, marine natural products, marine sponge, *Spongia* sp

## Abstract

Intensive study on the chemical components of a Korean marine sponge, *Spongia* sp., has led to the isolation of four new scalarane sesterterpenes, scalalactams A–D (**1**–**4**). Their chemical structures were elucidated from the analysis of spectroscopic data including 1D-and 2D-NMR as well as MS data. Scalalactams A–D (**1**–**4**) possess a scalarane carbon skeleton with a rare structural feature of a γ-lactam moiety within the molecules. Scalalactams A and B (**1** and **2**) have an extended isopropanyl chain at the lactam ring, and scalalactams C and D (**3** and **4**) possess a phenethyl group at the lactam ring moiety. Scalalactams A–D (**1**–**4**) did not show FXR antagonistic activity nor cytotoxicity up to 100 μM.

## 1. Introduction

Scalaranes are a class of sesterterpenes characterized by a 6/6/6/6-tetracyclic or 6/6/6/6/5-pentacyclic fused ring system and the conserved *trans*-configuration of A/B/C/D ring junctions [1]. Scalarane sesterterpenes are one of the numerically largest groups among the marine-derived sesterterpenes. Over two hundred scalarane sesterterpenes have been reported since the isolation of scalarin from *Cacospongia scalaris* in 1972 [2]. The structural diversity in scalarane sesterterpenes is mainly attributed to the various oxidation states at C-24 and C-25 [3]. However, in rare cases, mixed biogenetic products with nitrogen-containing moieties, which most likely arise from condensation with amino acids, have also been reported [4,5,6,7,8,9,10,11].

Despite the reported number of the Scalaranes, only eleven scalaranes with nitrogen-containing moieties have been reported so far [4,5,6,7,8,9,10,11]. After a long period following the initial isolation of pyrrole scalaranes in the 1970’s [4,5,6,7], six additional nitrogen-containing scalaranes with lactam moieties were later reported in the 21st century [8,9,10,11]. Considering the overall number of structurally similar scalaranes possessing a lactone ring, this number is extremely small.

Scalaranes are considered useful chemotaxonomic markers within sponges as they are isolated exclusively from the grazer nudibranchs [1]. Although the exact physiological or ecological purpose for which sponges produce scalaranes has not been clearly revealed, their antifeedant [12,13,14,15,16] and antifouling [17] activities give rise to the assumption that they are biosynthesized or stored for chemical defense [1]. They have also been investigated for biological activities such as cytotoxic [18,19,20,21], anti-tumor [22,23], antimicrobial [24,25,26], anti-inflammatory [27,28,29], platelet-aggregation inhibitory [30,31], and farnesoid X receptor (FXR) antagonistic [32,33] activities.

**Figure 1:**
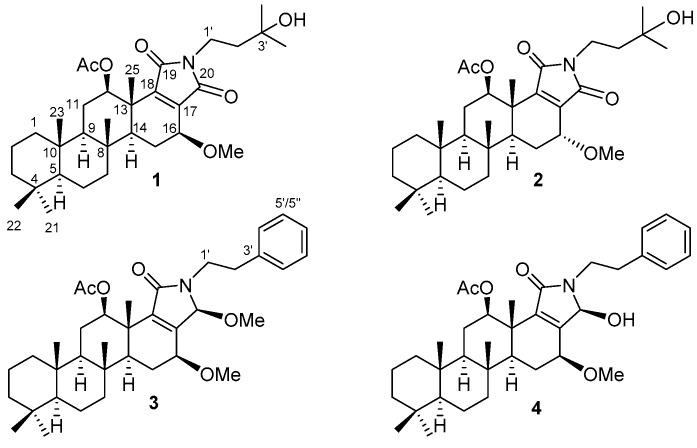
Scalalactams A–D (**1**–**4**).

As part of our investigation of ligands for nuclear receptors among marine natural products, we have studied specimens of the marine sponge, *Spongia* sp. In a previous study, we reported six new scalarane sesterterpenes with an antagonistic activity for farnesoid X-activated receptor (FXR) along with six known scalaranes from a marine sponge of the genus *Spongia* [32,33]. In the course of the investigation on minor components from the crude extract of this sponge to discover new secondary metabolites, we isolated four new scalarane sesterterpenes containing an unusual lactam moiety, scalalactams A–D (**1**–**4**) (Figure 1).

## 2. Results and Discussion

**Figure 2:**
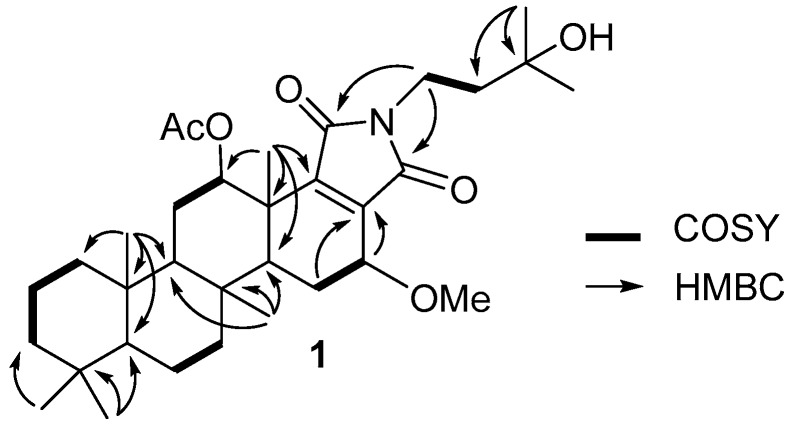
Key ^1^H-^1^H COSY and HMBC correlations of **1**.

The molecular formula of **1** was deduced as C_33_H_51_NO_6_, based on the ion of the protonated molecule at *m*/*z* 558.3788 [M + H]^+^ in HRFABMS. The ^1^H-NMR spectrum of **1** revealed the presence of two downfield methine protons [H-12 (*δ*_H_ 4.96, dd, *J* = 11.3, 4.8 Hz), and H-16 (*δ*_H_ 4.04, br d, *J* = 3.4 Hz)], together with two methylene systems [H-1′ (*δ*_H_ 3.56), and H-2′ (*δ*_H_ 1.68)]. The ^1^H-NMR spectrum of **1** also featured one acetyl group 12-OAc (*δ*_H_ 2.18), one methoxy group 16-OMe (*δ*_H_ 3.44) and seven methyl groups with all singlets [H_3_-21 (*δ*_H_ 0.84), H_3_-22 (*δ*_H_ 0.80), H_3_-23 (*δ*_H_ 0.83), H_3_-24 (*δ*_H_ 0.91), H_3_-25 (*δ*_H_ 1.21), H_3_-4′ (*δ*_H_ 1.24), and H_3_-5′ (*δ*_H_ 1.23)]. Interpretation of HSQC spectroscopic data of **1** (see Supplementary Materials) indicated nine methyl, nine methylene, five methine, and ten fully-substituted carbons. The structure of **1** was established from the interpretation of 2D spectroscopic data. ^1^H-^1^H COSY cross-peaks provided five spin systems [H-1/H-2/H-3, H-6/H-7, H-11/H-12, H-15/H-16, H-1’/H-2’]. Furthermore, the long-range HMBC correlations from two methyl singlets H_3_-21 and H_3_-22 to C-3, C-4, and C-5; from the methyl singlet H_3_-23 to C-1, C-5, C-9, and C-10; from the methyl singlet H_3_-24 to C-7, C-8, C-9, and C-14; from the methyl singlet H_3_-25 to C-12, C-13, C-14, and C-18 permitted the tetracyclic ring system of the scalarane carbon skeleton (Figure 2).

A 3′-hydroxyisopropanyl unit was established from the analysis of the ^1^H-^1^H COSY and HMBC spectroscopic data. A ^1^H-^1^H COSY cross-peak between H-1′ (*δ*_H_ 3.56) and H-2′ (*δ*_H_ 1.68), and long-range HMBC correlations from H_3_-4′ (*δ*_H_ 1.24) to C-2′ (*δ*_C_ 43.0), C-3′ (*δ*_C_ 69.9), and C-5′ (*δ*_C_ 28.9) provided the assignment of the 3′-hydroxy isopropanyl unit. Three-bond HMBC correlations from H-15 to C-17 (*δ*_C_ 138.8) and from H_3_-25 to C-18 (*δ*_C_ 150.9), and from H-1′ (*δ*_H_ 3.56) to C-19 and C-20 (both *δ*_C_ 169.1 not separable), suggested the presence of a pyrrole-2,5-dione moiety in the molecule. Lastly, the 3′-hydroxy isopropanyl unit which was connected through a nitrogen atom in the pyrrole-2,5-dione moiety, was determined from the chemical shifts of H-1′ (*δ*_H_ 3.56)/C-1′ (*δ*_C_ 33.5) and from the observation of three-bond HMBC correlations from H-1′ (*δ*_H_ 3.56) to C-19 and C-20 (both *δ*_C_ 169.1 not separable).

**Figure 3:**
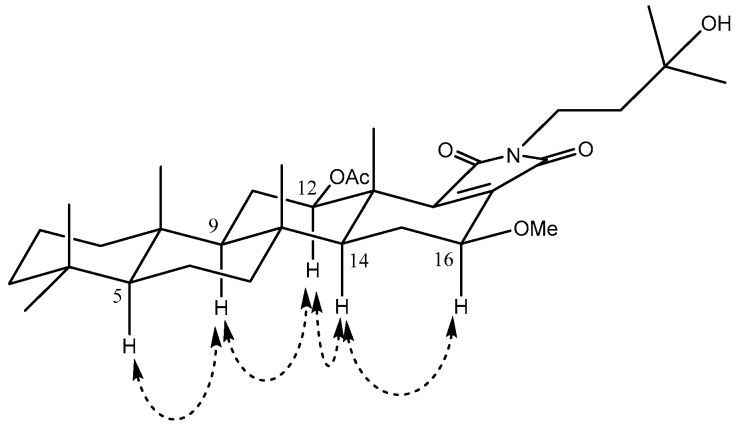
Key NOESY correlations of **1**.

The relative configurations of **1** were established from the analysis of coupling constants and NOESY spectra. NOESY cross-peaks [H-5/H-9/H-12/H-14/H-16] with large coupling constant of H-9 (*J* = 13.1 Hz) indicated the axial orientation of C-5, C-9, C-12, C-14, and C-16. The β-configuration of the acetyl group at C-12 was assigned by a coupling constant of H-12 (*J* = 11.3, 4.8 Hz) and NOESY correlations between H-12 and H-9, and between H-12 and H-14 [32,33]. A NOESY correlation between H-16 and H-14 also unambiguously suggested that the methoxy group at C-16 had the β-configuration in **1** (Figure 3).

The molecular formula of **2** was deduced as C_33_H_51_NO_6_ based on the ion detected at *m*/*z* 540.3690 [M + H − H_2_O]^+^ in HRFABMS. The ^1^H-NMR spectrum of **2** had similar features to that of **1**. Interpretation of 2D-NMR spectroscopic data indicated that the planar structure of **2** was the same as that of **1**. Analysis of the coupling constants and NOESY spectroscopic data of **2** also suggested that the relative configurations of **2** were almost identical to that of **1** except for C-16. A larger coupling constant value of H-16 (*δ*_H_ 4.12 dd, *J* = 9.5, 7.1 Hz) of compound **2** indicated the α-configuration of the methoxy group at C-16. Therefore, compound **2** was identified to be a 16-*epimer* of **1**.

The molecular formula of **3** was deduced as C_37_H_53_NO_5_ based on the protonated molecular ion at *m*/*z* 592.3995 [M + H]^+^ in HRFABMS. The IR spectrum showed the presence of an ester at 1737 cm^−1^ and 1238 cm^−1^. The ^1^H-NMR spectrum of **3** revealed the presence of five aromatic protons [H-4′/H-4′′ (*δ*_H_ 7.19, d, *J* = 7.3 Hz), H-5′/H-5′′ (*δ*_H_ 7.28, dd, *J* = 7.9, 7.3 Hz), H-6′ (*δ*_H_ 7.21, d, *J* = 7.9 Hz)], three downfield methine protons [H-12 (*δ*_H_ 4.97, dd, *J* = 11.1, 4.6 Hz), H-16 (*δ*_H_ 3.75, overlap with H-1′), and H-20 (*δ*_H_ 5.10, s)], together with two downfield methylene systems [H-1′ (*δ*_H_ 3.75, overlap with H-16)/(*δ*_H_ 3.20, q, *J* = 8.0 Hz), and H-2′ (*δ*_H_ 2.83, m)]. The ^1^H-NMR spectrum of **3** also displayed one acetyl group 12-OAc (*δ*_H_ 2.18, s), and two methoxy protons [16-OMe (*δ*_H_ 3.35, s) and 20-OMe (*δ*_H_ 2.90, s)], five methyl singlets [H_3_-21 (*δ*_H_ 0.84), H_3_-22 (*δ*_H_ 0.80), H_3_-23 (*δ*_H_ 0.82), H_3_-24 (*δ*_H_ 0.91), and H_3_-25 (*δ*_H_ 1.22)]. Analysis of HSQC spectroscopic data of **3** indicated eight methyl, nine methylene, eleven methine, and nine fully-substituted carbons. The structure of **3** was established from the interpretation of ^1^H-^1^H COSY and HMBC spectroscopic data. ^1^H-^1^H COSY cross-peaks [H-1/H-2/H-3, H-6/H-7, H-9/H-11/H-12, H-15/H-16, H-1′/H-2′] provided two sets of three-carbon and three sets of two-carbon units. ^1^H-^1^H COSY correlations of H-4′/H-4′′and H-6′ to H-5′/H-5′′ also permitted a phenyl group. The phenyl group was further extended with two carbon units from the observation of long-range HMBC correlations from the methylene protons H-2′ to carbons C-3′, and C-4′/C-4′′. The scalarane moiety was established from the interpretation of HMBC spectroscopic data. In particular, the long-range HMBC correlations from two methyl singlets H_3_-21 and H_3_-22 to C-3, C-4, and C-5; from the methyl singlet H_3_-23 to C-1, C-5, C-9, and C-10; from the methyl singlet H_3_-24 to C-7, C-8, C-9, and C-14; from the methyl singlet H_3_-25 to C-12, C-13, C-14, and C-18; from a methine proton H-20 to 20-OMe, C-17, C-18, and C-19 allowed the scalarane carbon skeleton to be established. Additionally, the chemical shifts difference of C-17 (*δ*_C_ 148.0) and C-18 (*δ*_C_ 146.1) secure the position of the carbonyl at C-19 [34]. Unfortunately, no HMBC correlations from H-1′ to carbons C-19/C-20 were observed. However, based on the chemical shifts of H-1′ (*δ*_H_ 3.20, q, *J* = 8.0 Hz)/(*δ*_H_ 3.75, m) and C-1′ (*δ*_C_ 41.6), the only plausible structure for **3** was that a phenethyl unit and scalarane moiety connected through a nitrogen atom. A three-bond HMBC correlation from H-12 (*δ*_H_ 4.97) to 12-OAc (*δ*_C_ 172.0) indicated an acetyl group at position C-12. In a similar fashion, three-bond HMBC correlations from methyl singlets 16-OMe to C-16 (*δ*_C_ 70.0), and from 20-OMe to C-20 (*δ*_C_ 85.0) suggested that two methoxy groups were located at C-16 and C-20, respectively.

Relative configurations of **3** were determined through analysis of the coupling constants and NOESY spectroscopic data. NOESY cross-peaks [H-5/H-9/H-12/H-14/H-16] with large coupling constant of H-9 (*J* = 12.3 Hz) indicated the axial orientation of C-5, C-9, C-12, C-14, and C-16. NOESY cross-peaks [H_3_-22/H-3β (*δ*_H_ 1.35), H_3_-23/H-1β or H-2β (*δ*_H_ 1.59 or 1.61, merged in NOESY spectra), H_3_-24/H-7β (*δ*_H_ 1.79), H_3_-24/H_3_-25] suggested the β-congifuration of C-22, C-23, C-24, and C-25 [9]. NOESY correlations between H-16 and H-14 suggested that the methoxy group at C-16 had the β-configuration. The coupling constant of H-12 (*J* = 11.1 Hz) also indicated that H-12 had an axial orientation. The β-configuration of the methoxy group at C-20 was determined from the observation of a NOESY correlation between H-16 (*δ*_H_ 3.75) and H-20 (*δ*_H_ 5.10).

The molecular formula of **4** was deduced as C_36_H_51_NO_5_ based on the protonated peak at *m*/*z* 578.3855 [M + H]^+^ in HRFABMS. The ^1^H-NMR spectrum of **4** was almost identical to that of **3** except for the absence of one methyl singlet and the presence of an exchangeable proton. The observation of ^1^H-^1^H COSY correlations between 20-OH (*δ*_H_ 1.49) and H-20 (*δ*_H_ 5.05) indicated that **4** had a hydroxy group at C-20 instead of a methoxy group. Interpretation of 2D-NMR spectroscopic data allowed the planar structure of **4** to be assigned as shown.

Relative stereochemistry of **4** was established by analysis of coupling constants and NOESY spectroscopic data. Similar NOESY cross-peaks were observed with compound **3** which suggested the same orientation of the carbons. A large-magnitude coupling constant for H-12 (*J* = 11.1, 4.7 Hz) suggested the β-configuration of the acetyl group at C-12, while a NOESY correlation between H-14 and H-16 indicated the β-configuration of the methoxy group at C-16, respectively. The β-configuration of the hydroxy group at C-20 was also assigned from the observation of NOESY correlations between 20-OH (*δ*_H_ 1.47) and 16-OMe, and between H-20 and H-16. There is possibility that these new isolates are methylated artifact based on the presence of *O*-methoxy group at C-16 and C-19. It wasn’t possible to address this as the supply of the raw sponge material was limited.

Previously isolated scalaranes from a *Spongia* sp. showed farnesoid X-activated receptor (FXR) antagonistic activity and cytotoxicity against a CV-1 monkey kidney cell line [32,33]. FXR is a ligand-dependent nuclear receptor that controls lipoprotein metabolism and cholesterol homeostasis. The scalaranes containing a lactone moiety showed moderate IC_50_ values in an FXR cell transactivation assay from 2.4 to 81.1 μM. However, scalalactams A–D (**1**–**4**) did not display any significant FXR antagonistic activity up to 100 μM. This result suggests that the lactone ring moiety within the scalarane class of natural products could be an important pharmacophore for FXR antagonistic activity. The loss of cytotoxicity of scalalactams A–D against the CV-1 cell line up to 100 μM also confirms the importance of the lactone ring moiety. Unfortunately, additional biological evaluations were not possible due to the small amounts of the compounds isolated.

There have been numerous efforts to obtain hybrid systems based on steroid frames combined with amino acids, called steroid-amino acid hybrids, to explore diverse physical, chemical, and biological properties [35,36,37]. Scalalactams A–D, which are scalaranes containing a γ-lactam ring, are good examples that steroid-amino acid hybrid tactics can be employed for the scalarane class of natural products [38].

## 3. Materials and Methods

### 3.1. General Information

Optical rotations were measured on an Autopol III polarimeter #A7214 (Rudolph Research Analytical, Hackettstown, NJ, USA) equipped with a 5 cm cell. Infrared spectra were recorded on a NICOLET 5700 spectrometer (Thermo Electron Corp, Waltham, MA, USA) and ultraviolet spectra were also recorded on a Scinco UVS-2100 instrument (Scinco, Seoul, Korea). ^1^H- and ^13^C-NMR spectra were recorded on an Avance DPX-600 spectrometer (Bruker, Billerica, MA, USA). FAB-MS were measured on a JMS-AX505WA mass spectrometer (JEOL, Tokyo, Japan). Solvents used in partitioning were first grade products of Dae Jung & Metals Co. (Siheung, Korea). HPLC grade solvents from Brudick & Jackson (Muskegon, MI, USA) were used in adsorption chromatography, TLC and HPLC. Younglin SDV 30 plus HPLC’s with Younglin M 720 UV detectors were used for isolation of compounds (YL Instruments, Anyang, Korea). NMR solvents were obtained from Cambridge Isotope Laboratories (CIL), Inc. (Tewksbury, MA, USA).

### 3.2. Animal Material

A marine sponge specimen was collected at a depth of 10 m in the South Sea near Tong-Yong City, Korea. The sponge was immediately frozen by packing with dry ice and then stored at −18 °C until further processing. The sponge was identified as a species of the genus *Spongia*. The color of the sponge is typically brown. The shape is compact and round. The skeleton is comprised of a tightly meshed system and its consistency is compressible. A voucher specimen was deposited at the Center for Marine Natural Products and Drug Discovery, Seoul National University, Seoul, Korea.

### 3.3. Extraction and Isolation

The frozen sponge (23.0 kg, wet weight) was extracted three times with 50% MeOH in DCM. These extracts were combined and partitioned three times between hexanes and MeOH. Then the MeOH-soluble layer was further partitioned between ethylacetate (EtOAc, 10 g) and water three times. The EtOAc-soluble layer was subjected to silica flash chromatography using stepped gradient mixtures of EtOAc and hexanes as eluents to provide 21 fractions. Fraction three was further separated by using repeated reverse-phased HPLC (Optimapak, 250 × 10 mm, 5 μm, 100 Å, UV = 210 nm), eluting with 85% acetonitrile in H_2_O to afford **1** (0.6 mg), **2** (0.5 mg), **3** (0.6 mg), and **4** (0.4 mg), as colorless oils.

*Scalalactam A (**1**):* [α]_D_^25^ + 3° (c 0.002, CHCl_3_); UV *λ*max (log *ε*) 210 (3.83), 232 (2.10); IR (KBr) ν max: 3393, 1761, 1682, 1235 cm^−1^; ^1^H-, ^13^C-, and 2D-NMR data, Table 1; HRFABMS *m*/*z* 558.3788 [M + H]^+^ (calcd. for C_33_H_52_NO_6_^+^, 558.3789).

*Scalalactam B (**2**):* [α]_D_^25^ + 5° (c 0.002, CHCl_3_); UV *λ*max (log *ε*) 210 (3.83), 232 (2.09); IR (KBr) ν max: 3391, 1763, 1681, 1233 cm^−1^; ^1^H-, ^13^C-, and 2D-NMR data, Table 1; HRFABMS *m*/*z* 540.3690 [M + H − H_2_O]^+^ (calcd. for C_33_H_50_NO_5_^+^, 540.3684).

*Scalalactam C (**3**):* [α]_D_^25^ + 1° (c 0.002, CHCl_3_); UV *λ*max (log *ε*) 210 (3.83), 254 (2.12) nm; IR (KBr) ν max: 3392, 1760, 1683, 1238 cm^−1^; ^1^H- and ^13^C-NMR data, Table 2; HRFABMS *m*/*z* 592.3995 [M + H]^+^ (calcd. for C_37_H_54_NO_5_^+^, 592.3997).

*Scalalactam D (**4**):* [α]_D_^25^ + 6° (c 0.002, CHCl_3_); UV *λ*max (log *ε*) 210 (3.83), 254 (2.11) nm; IR (KBr) ν max: 3392, 1765, 1681, 1235 cm^−1^; ^1^H-and ^13^C-NMR data, Table 2; HRFABMS *m*/*z* 578.3855 [M + H]^+^ (calcd. for C_36_H_52_NO_5_^+^, 578.3840).

### 3.4. Co-transfection Assay

CV-1 cells were seeded in 96-well plates in Dulbecco’s modified Eagle’s medium (GIBCO) supplemented with 10% lipid-depleted fetal bovine serum in humidified air containing 5% CO_2_ at 37 °C for 24 h. Transient cotransfection with pCMX-hFXR, pCMX-β-GAL and Tk-(EcRE)6-LUC were carried out using SuperFect (Qiagen, Venlo, Netherlands), according to the manufacturer’s instructions. After 24 h incubation, cotransfected cells were treated with a control vehicle (DMSO), or the indicated compounds, for the FXR antagonist test in the presence of 50 μM chenodeoxycholic acid (CDCA, a natural ligand for FXR). Cells were harvested at 24 h, and luciferase activities were assayed as described [32,33]. Luciferase activities were normalized to the β-galactosidase activity expressed from the control plasmid pCMX-β-GAL. Each transfection was performed in triplicate.

### 3.5. Cytotoxicity Assay

CV-1 cells were seeded in 96-well plates in DMEM supplemented with 10% fetal bovine serum in humidified air containing 5% CO_2_ at 37 °C. After 24 h incubation, indicated compounds were administrated at various concentrations up to 100 μM. Cells were harvested at 24 h and were incubated with 1 mg/mL 3-4,5-dimethylthiazol-(2-yl)-2,5-diphenyltetrazolium bromide (MTT) solution for 1 h at 37 °C. DMSO was added to each well in order to dissolve the produced purple formazan crystals, and the absorbance of each well was measured at 450 nm using an ELISA reader. The experiment was carried out in triplicate and repeated three times.

## Figures and Tables

**Table 1:** NMR spectroscopic data of **1** and **2** in CDCl_3_ at 600 MHz.

		1				2
No.	*δ*_C_, m ^*a*^	*δ*_H_, m ^*a*^, *J* (Hz)	^1^H-^1^H COSY	HMBC (10 Hz)	*δ*_C_, m ^*a*^	*δ*_H_, m ^*a*^, *J* (Hz)
1	39.3, t	0.83 ^*c*^; 1.61 ^*c*^		2, 5, 9, 10	39.3, t	0.83, m; 1.61, m
2	18.2, t	1.40, m; 1.58, m			18.3, t	1.40, m; 1.59, m
3	41.9, t	1.11, m; 1.35, m	3		41.9, t	1.13, m; 1.35, m
4	33.1, s		2	21, 22	33.1, s	
5	56.5, d	0.81*^c^*			56.5, d	0.78, dd, *J* = 12.3, 1.5
6	18.5, t	1.38, m; 1.57, m			18.5, t	1.38, m; 1.58, m
7	42.1, t	0.98, m; 1.77, m			41.5, t	0.94, m; 1.84, m
8	39.1, s				39.1, s	
9	58.0, d	1.05, dd, *J* = 13.1, 2.3	11	5, 7, 8, 11, 12, 14, 23, 24	58.0, d	0.94, dd, *J* = 12.1, 2.3
10	37.1, s				37.1, s	
11	23.8, t	1.56, m; 1.77, m	9, 12	9, 12, 10, 13	24.4, t	1.55, m; 1.74, m
12	75.4, d	4.96, dd, *J* = 11.3, 4.8	11		75.4, d	4.88, dd, *J* = 11.1, 4.6
13	43.5, s				42.8, s	
14	49.8, d	1.61, m	15	7, 9, 12, 15, 16, 18, 24, 25	54.7, d	1.12, m
15	22.2, t	1.48, m; 2.08, br d, *J* = 14.0	14, 16		24.4, t	1.75, d, *J* = 12.8; 2.28, dd, *J* = 12.8, 7.1
16	68.1, d	4.04, br d, *J* = 4.5	15		74.1, d	4.12, dd, *J* = 9.5, 7.1
17	138.8, s				140.8, s	
18	150.9, s				150.5, s	
19	169.1 ^*b*^				169.4^b^	
20	169.1 ^*b*^				169.4^b^	
21	33.2, q	0.84, s		3, 4, 5, 22	33.2, q	0.84, s
22	21.8, q	0.80, s		3, 4, 5, 21	21.4, q	0.80, s
23	16.3, q	0.83, s		1, 5, 9, 10	16.3, q	0.82, s
24	16.9, q	0.91, s		7, 8, 9, 14	18.2, q	0.92, s
25	16.1, q	1.21, s		12, 13, 14, 18	17.0, q	1.32, s
12-OAc	171.8, s				171.8, s	
	22.0, q	2.18, s			22.2, q	2.17, s
16-OMe	58.7, q	3.44, s			59.1, q	3.55, s
1′	33.5, t	3.57, t, *J* = 7.1			33.5, t	3.56, t, *J* = 6.8
2′	43.0, t	1.68, t, *J* = 7.1			41.0, t	1.68, q, *J* = 6.8
3′	69.9, s				69.9, s	
4′	28.9, q	1.24, s			28.8, q	1.24, s
5′	28.9, q	1.23, s			28.8, q	1.22, s

*^a^* Multiplicity was determined by analysis of 2D spectroscopic data. *^b^* Chemical shifts of these two carbons are overlapped. *^c^* Multiplicity was not determined due to the signal overlap.

**Table 2:** ^1^H- and ^13^C-NMR spectroscopic data of **3** and **4** in CDCl_3_ at 600 MHz.

		3		4
No.	*δ*_C_, m ^*a*^	*δ*_H_, m ^*a*^, *J* (Hz)	*δ*_C_, m ^*a*^	*δ*_H_, m ^*a*^, *J* (Hz)
1	40.0, t	0.84, m; 1.61, m	40.0, t	0.83, m; 1.63, dt, *J* = 12.5, 1.5
2	18.4, t	1.41, m; 1.59, m	18.6, t	1.41, m; 1.59, m
3	41.9, t	1.11, m; 1.36, m	42.2, t	1.17, m; 1.35, m
4	33.4, s		33.3, s	
5	56.5, d	0.84 ^*b*^	56.5, d	0.83 ^*b*^
6	18.6, t	1.38, m; 1.56, m	18.5, t	1.38, m; 1.53, m
7	41.3, t	0.98, m; 1.79, dt, *J* = 12.3, 1.5	41.5, t	0.94, m; 1.79, dt, *J* = 12.3, 1.5
8	37.0, s		36.8, s	
9	57.6, d	1.03, d, *J* = 12.3	57.8, d	1.01, br d, *J* = 12.1
10	37.2, s		39.8, s	
11	25.0, t	1.55, m; 1.77, m	24.9, t	1.55, m; 1.75 ^*b*^
12	75.5, d	4.97, dd, *J* = 11.1, 4.6	75.6, d	4.95, dd, *J* = 11.1, 4.7
13	42.6, s		42.0, s	
14	50.0, d	1.47, m	50.2, d	1.43, m
15	21.8, t	1.54, m; 2.08, d, *J* = 13.5	22.1, t	1.42, m; 2.06, d, *J* = 13.5
16	70.0, d	3.75 ^*b*^	69.6, d	3.92, d, *J* = 3.4
17	148.0, s		150.2, s	
18	146.1, s		144.0, s	
19	168.2, s		167.5, s	
20	85.0, d	5.10, s	80.4, d	5.05, d, *J* = 10.3
21	33.2, q	0.84, s	33.2, q	0.82, s
22	21.8, q	0.80, s	21.8, q	0.79, s
23	16.3, q	0.82, s	16.6, q	0.82, s
24	17.0, q	0.91, s	17.4, t	0.91, s
25	16.2, q	1.22, s	15.9, q	1.20, s
12-OAc	172.0, s		171.8, s	
	21.1, q	2.18, s	21.4, q	2.17, s
16-OMe	57.5, q	3.35, s	57.3, q	3.34, s
20-OMe	49.1, q	2.90		
1′	41.6, t	3.20, q, *J* = 8.0; 3.75 ^*b*^	41.9, t	3.48, q, *J* = 8.0; 3.65, q, *J* = 8.0
2′	34.9, t	2.83, m	34.9, t	2.85, m
3′	139.0, s		139.6, s	
4′, 4′′	128.7, d	7.19, d, *J* = 7.3	128.7, d	7.21, d, *J* = 7.9,
5′, 5′′	128.5, d	7.28, dd, *J* = 7.9, 7.3	128.5, d	7.29, dd, *J* = 7.9, 7.9
6′	126.0, d	7.21, d, *J* = 7.9	126.0, d	7.19, d, *J* = 7.9
20-OH				1.49, d, *J* = 10.3

*^a^* Multiplicity was determined by analysis of 2D spectroscopic data. *^b^* Multiplicity was not determined due to the signal overlap.

## Supplementary Materials

The following are available online, The ^1^H, ^1^H-^1^H COSY, HSQC, HMBC, NOESY NMR spectroscopic data of scalalactams A–D (**1**–**4**).
