# Supplementary material for: Scalalactams A–D, Scalarane Sesterterpenes with a γ-Lactam Moiety from a Korean Spongia Sp. Marine Sponge
Source: Molecules. 2018 Dec 3;23(12):3187. doi: 10.3390/molecules23123187 (PMC6321518; doi:10.3390/molecules23123187)

Supporting Information for

Article

Scalalactams A-D, Scalarane sesterterpenes with a γ-lactam moiety from a Korean marine sponge *Spongia* sp.

Inho Yang [1,†], Jusung Lee [2,†], Jihye Lee [2], Dongyup Hahn [3,4], Jungwook Chin [5], Dong Hwan Won [2], Jaeyoung Ko [6], Hyukjae Choi [7], Ahreum Hong [8], Sang-Jip Nam [9,*], and Heonjoong Kang [2,10,*]

[1] Department of Convergence Study on the Ocean Science and Technology, Korea Maritime and Ocean University, Busan 49112, Korea
[2] The Center for Marine Natural Products and Drug Discovery, School of Earth and Environmental Science, Seoul National University, NS-80, Seoul 08826, Korea
[3] School of Food Science and Biotechnology, Kyungpook National University, Daegu 41566, Korea
[4] Institute of Agricultural Science & Technology, Kyungpook National University, Daegu 41566, Korea
[5] New Drug Development Center, Daegu-Gyeongbuk Medicinal Innovation Foundation, Daegu, 41061, Korea
[6] Skin Research Division, Amorepacific R&D Unit, Yongin 449-729, Korea
[7] College of Pharmacy, Yeungnam University, Gyeongsan 38541, Korea
[8] Graduate School of Industrial Pharmaceutical Sciences, Ewha Womans University, Seoul 03760, Korea
[9] Department of Chemistry and Nanoscience, Ewha Womans University, Seoul 03760, Korea
[10] Research Institute of Oceanography, Seoul National University, NS-80, Seoul 08826, Korea
[†] These authors contributed equally to this work.
* Correspondence: sjnam@ewha.ac.kr (S. Nam); hjkang@snu.ac.kr (H. Kang)

Contents

Figure S1. ^{1}H NMR spectrum of scalalactam A (**1**) in $CDCl_3$ (600 MHz) …………… S1

Figure S2. COSY spectrum of scalalactam A (**1**) in $CDCl_3$ (600 MHz) ……………… S2

Figure S3. HSQC spectrum of scalalactam A (**1**) in $CDCl_3$ (600 MHz) ……………... S3

Figure S4. HMBC spectrum of scalalactam A (**1**) in $CDCl_3$ (600 MHz) …………….. S4

Figure S5. NOESY spectrum of scalalactam A (**1**) in $CDCl_3$ (600 MHz) ……………. S5

Figure S6. ^{1}H NMR spectrum of scalalactam B (**2**) in $CDCl_3$ (600 MHz) …………… S6

Figure S7. COSY spectrum of scalalactam B (**2**) in $CDCl_3$ (600 MHz) ……………... S7

Figure S8. HSQC spectrum of scalalactam B (**2**) in $CDCl_3$ (600 MHz) ……………... S8

Figure S9. HMBC spectrum of scalalactam B (**2**) in $CDCl_3$ (600 MHz) …………….. S9

Figure S10. NOESY spectrum of scalalactam B (**2**) in $CDCl_3$ (600 MHz) ………… S10

Figure S11. ^{1}H NMR spectrum of scalalactam C (**3**) in $CDCl_3$ (600 MHz) ………… S11

Figure S12. COSY spectrum of scalalactam C (**3**) in $CDCl_3$ (600 MHz) ……………. S12

Figure S13. HSQC spectrum of scalalactam C (**3**) in $CDCl_3$ (600 MHz) …………… S13

Figure S14. HMBC spectrum of scalalactam C (**3**) in $CDCl_3$ (600 MHz) …………… S14

Figure S15. NOESY spectrum of scalalactam C (**3**) in $CDCl_3$ (600 MHz) ………… S15

Figure S16. ^{1}H NMR spectrum of scalalactam D (**4**) in $CDCl_3$ (600 MHz) ………… S16

Figure S17. COSY spectrum of scalalactam D (**4**) in $CDCl_3$ (600 MHz) ……………. S17

Figure S18. HSQC spectrum of scalalactam D (**4**) in $CDCl_3$ (600 MHz) ……………. S18

Figure S19. HMBC spectrum of scalalactam D (**4**) in $CDCl_3$ (600 MHz) …………… S19

Figure S20. NOESY spectrum of scalalactam D (**4**) in $CDCl_3$ (600 MHz) ………….. S20

Figure S21. FAB-MS Spectrum of **1**…………………………………………………. S21

Figure S22. FAB-MS Spectrum of **2**…………………………………………………. S22

Figure S23. FAB-MS Spectrum of **3**…………………………………………………. S23

Figure S24. FAB-MS Spectrum of **4** S24

Figure S1. ^1H NMR spectrum of scalalactam A (**1**) in $CDCl_3$ (600 MHz)

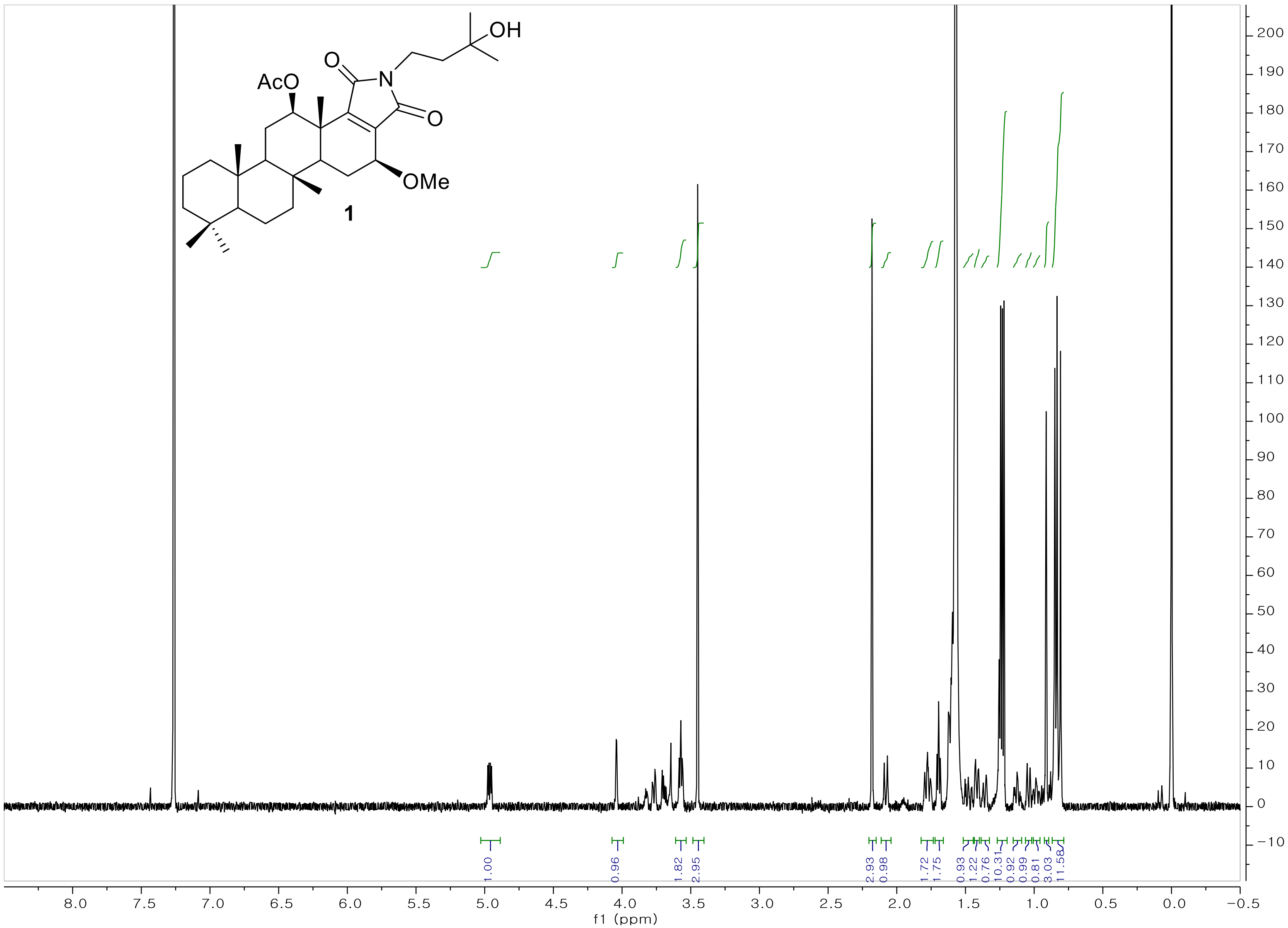

Figure S2. COSY spectrum of scalalactam A (**1**) in $CDCl_3$ (600 MHz)

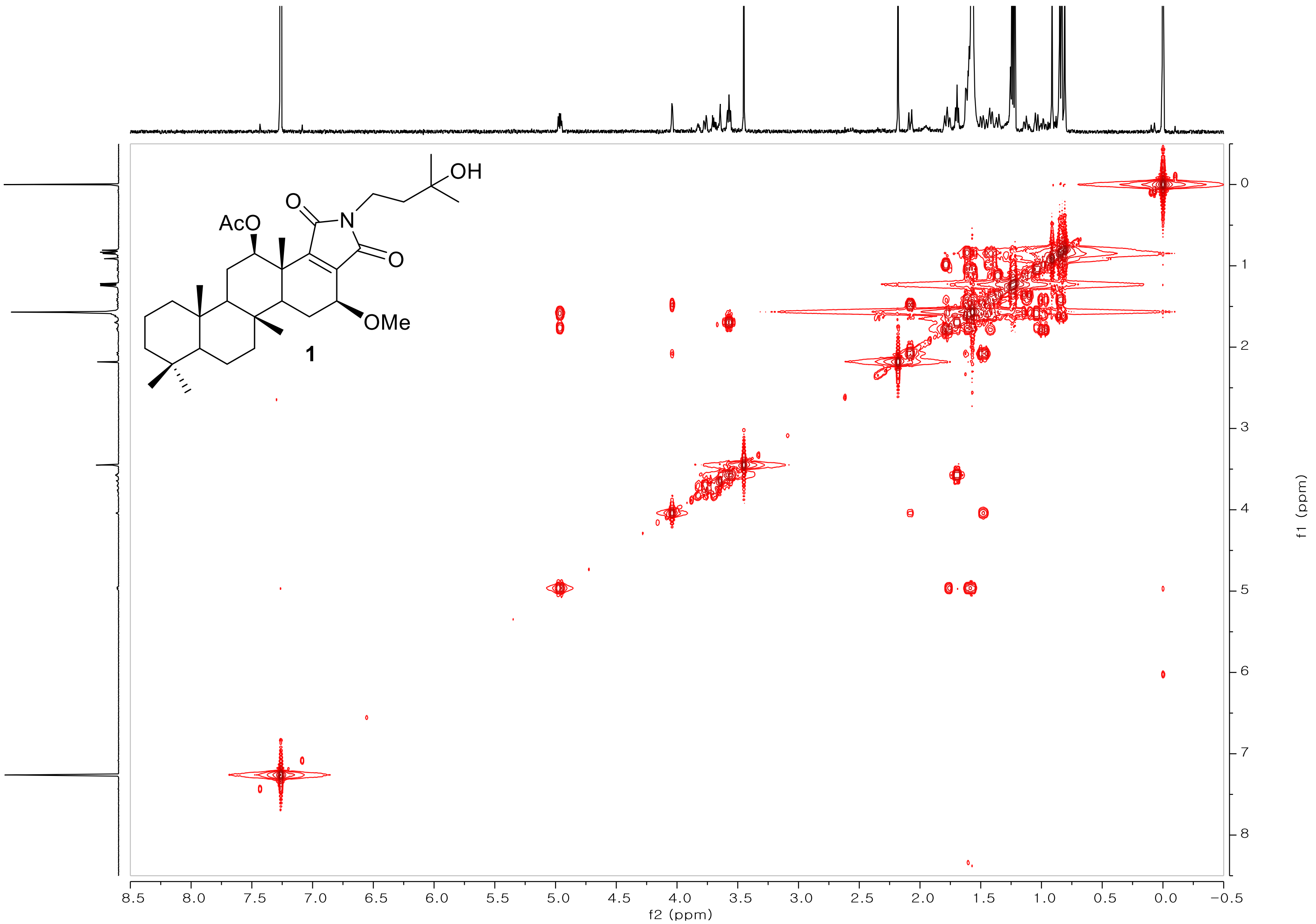

Figure S3. HSQC spectrum of scalalactam A (**1**) in $CDCl_3$ (600 MHz)

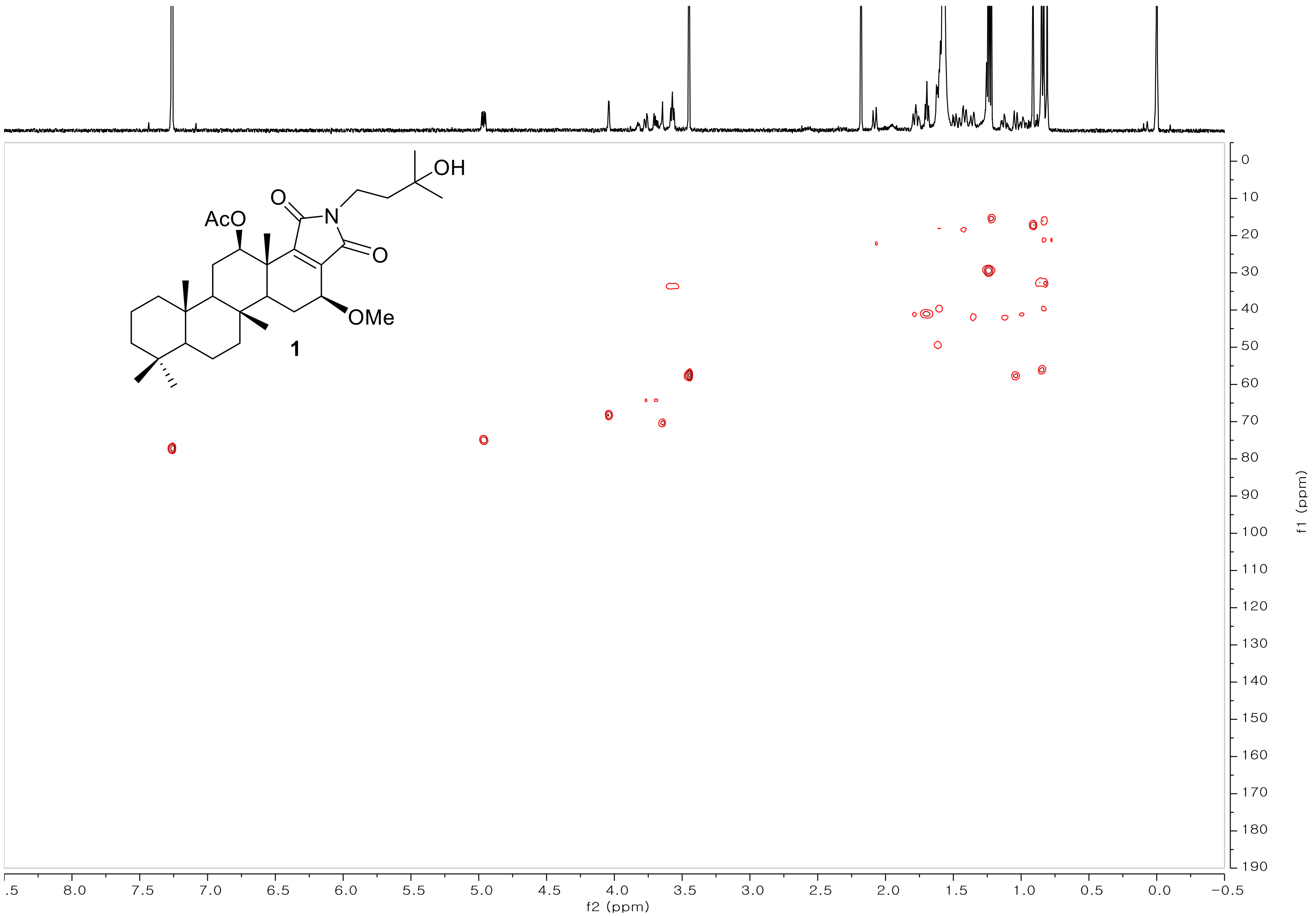

Figure S4. HMBC spectrum of scalalactam A (**1**) in $CDCl_3$ (600 MHz)

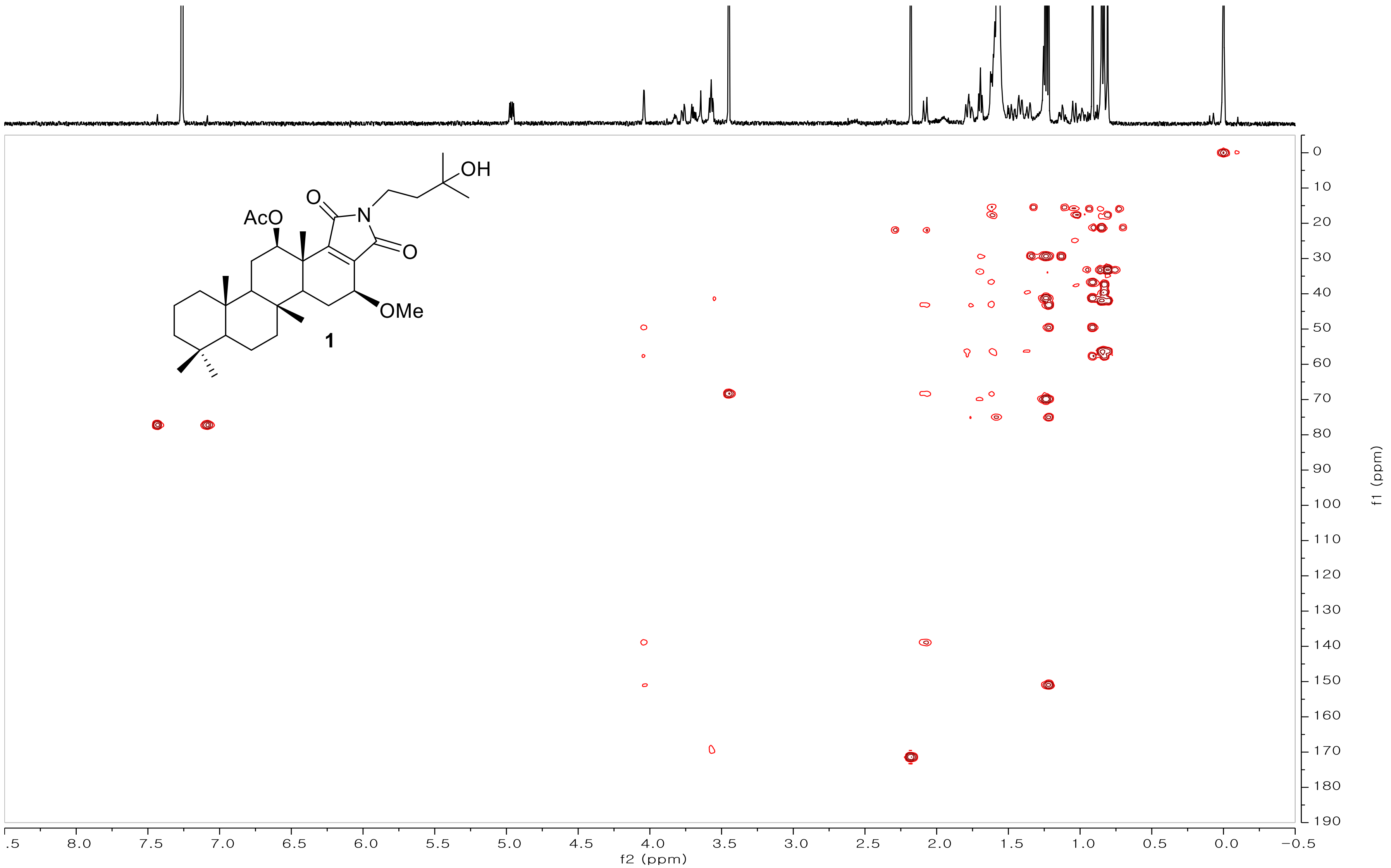

Figure S5. NOESY spectrum of scalalactam A (**1**) in $CDCl_3$ (600 MHz)

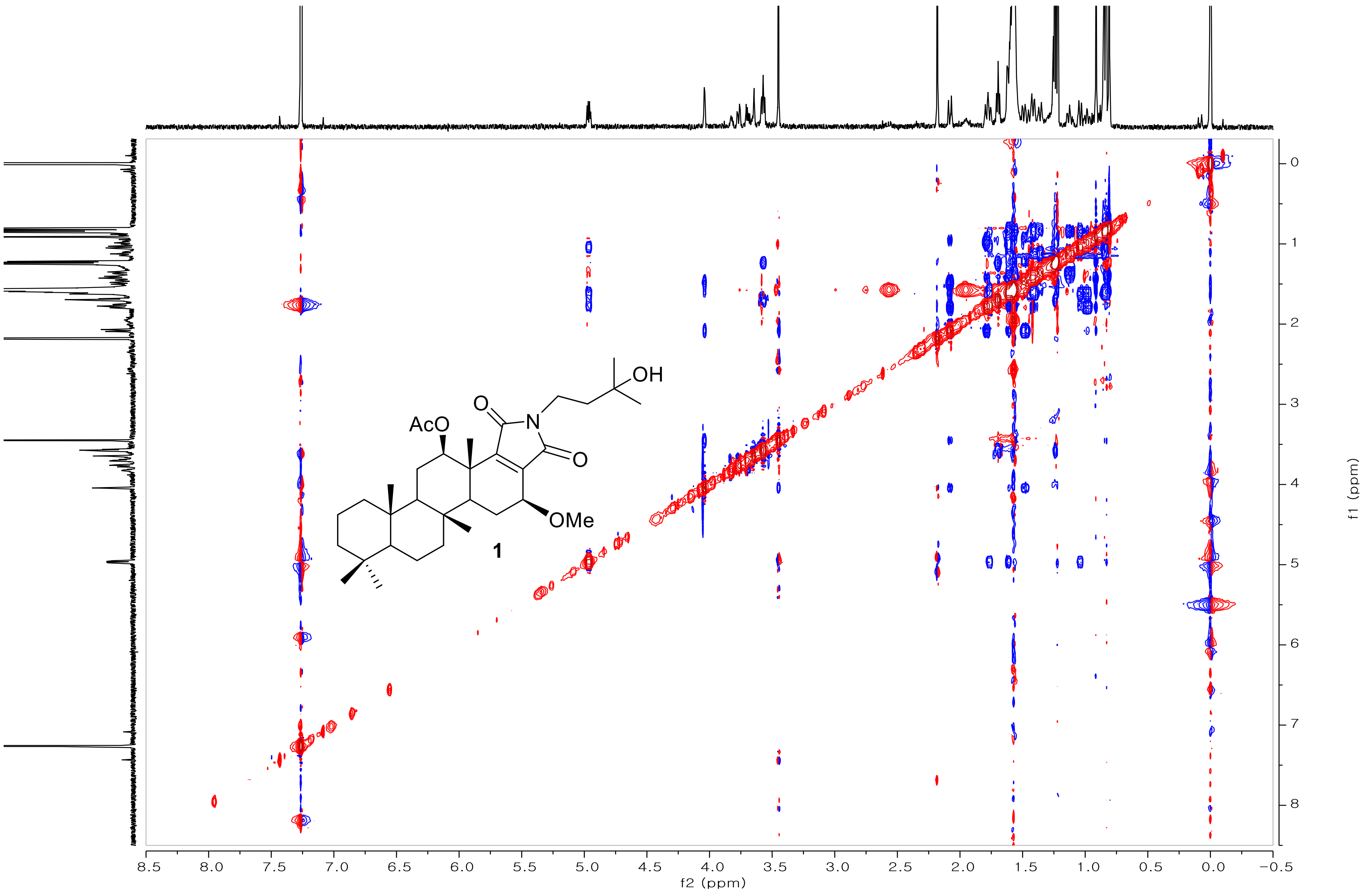

Figure S6. ^{1}H NMR spectrum of scalalactam B (**2**) in $CDCl_3$ (600 MHz)

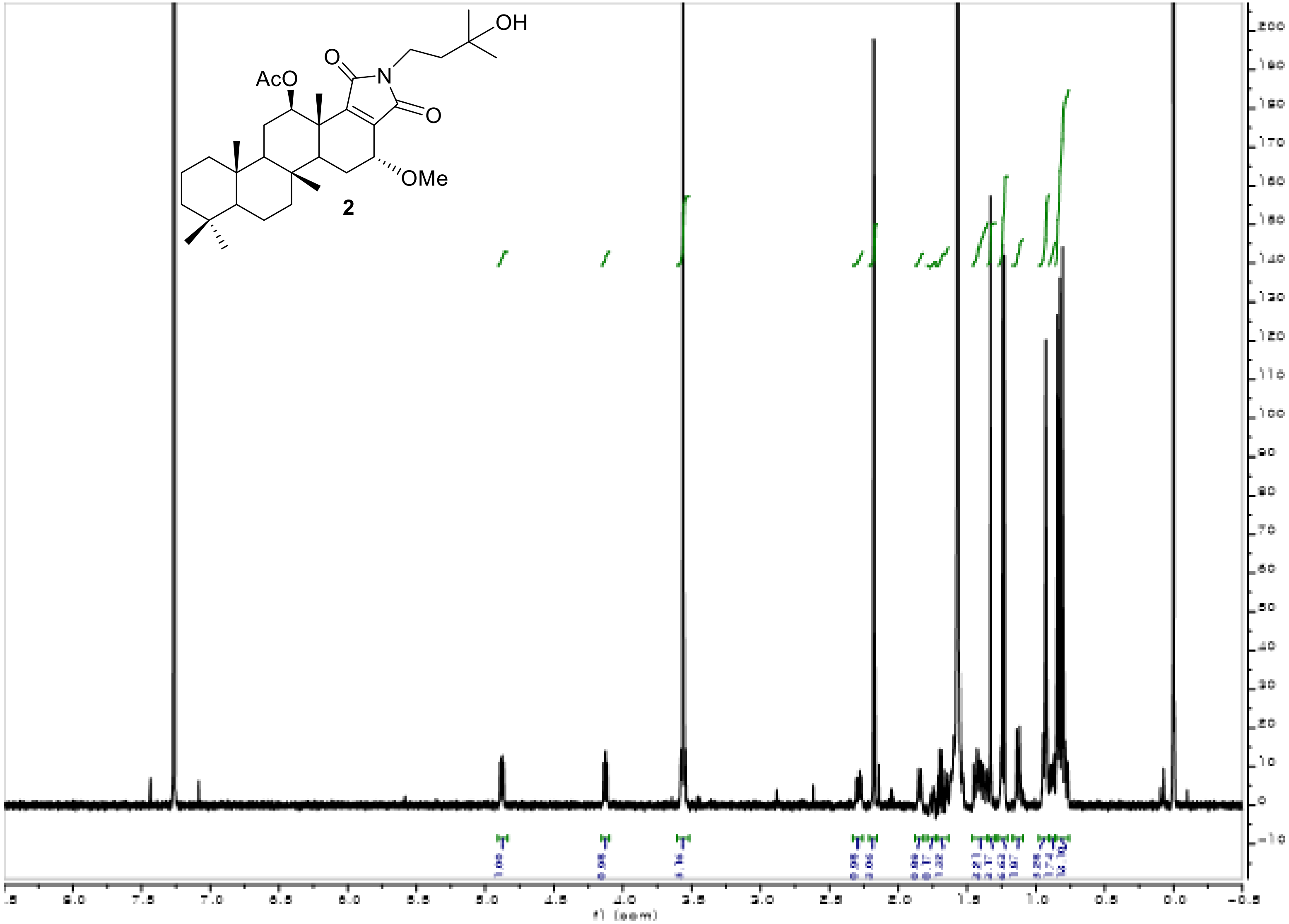

Figure S7. COSY spectrum of scalalactam B (**2**) in $CDCl_3$ (600 MHz)

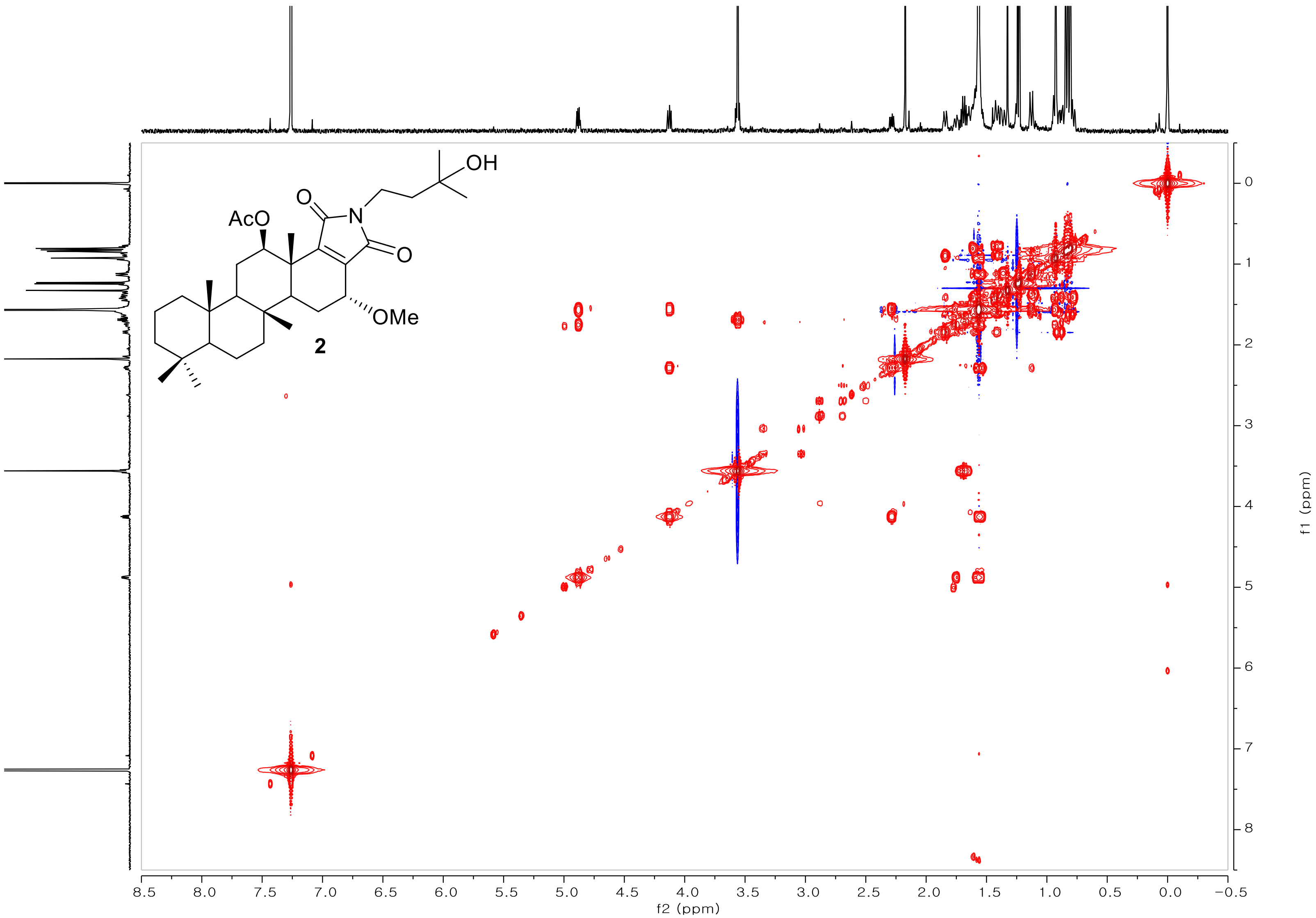

Figure S8. HSQC spectrum of scalalactam B (**2**) in $CDCl_3$ (600 MHz)

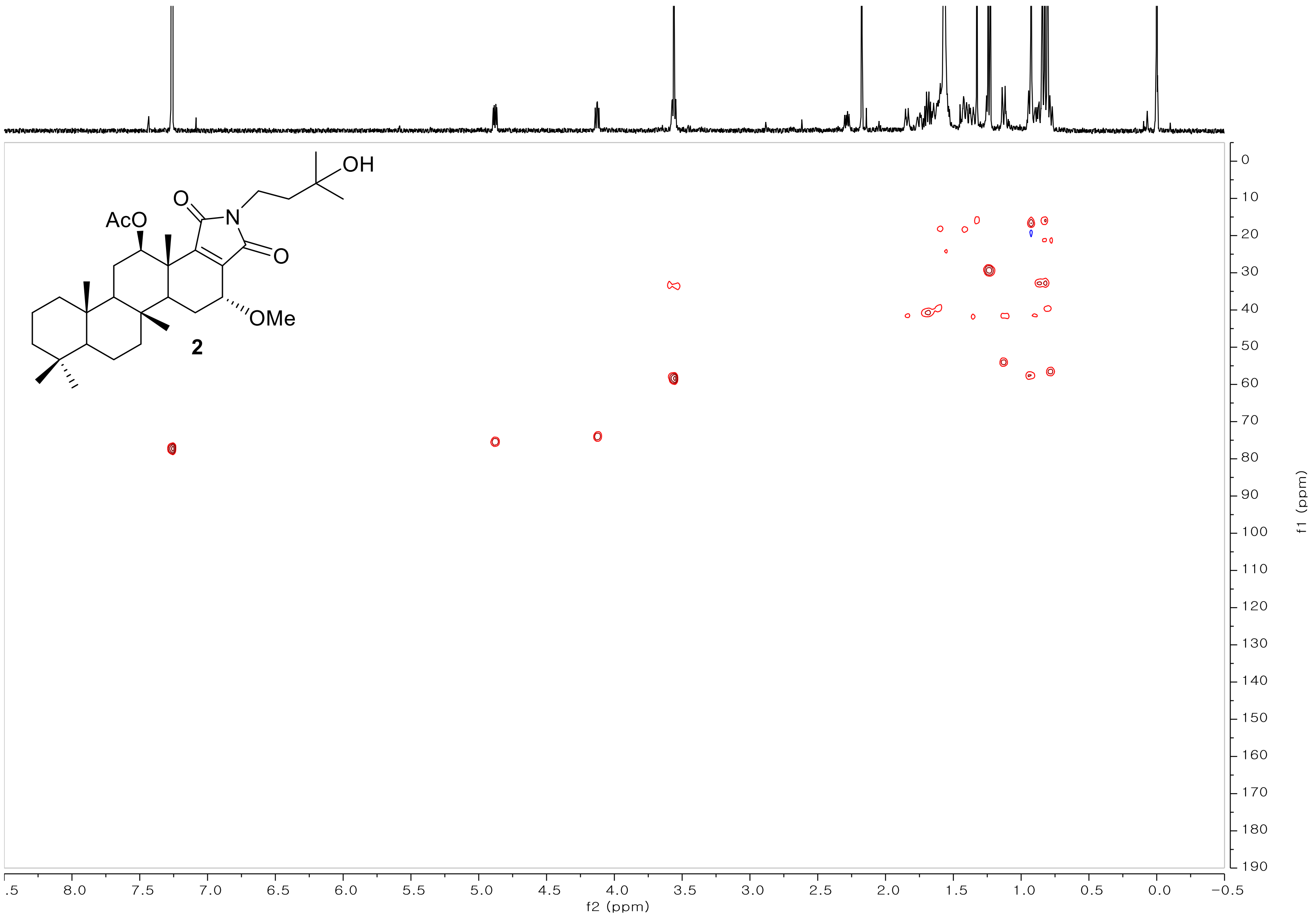

Figure S9. HMBC spectrum of scalalactam B (**2**) in $CDCl_3$ (600 MHz)

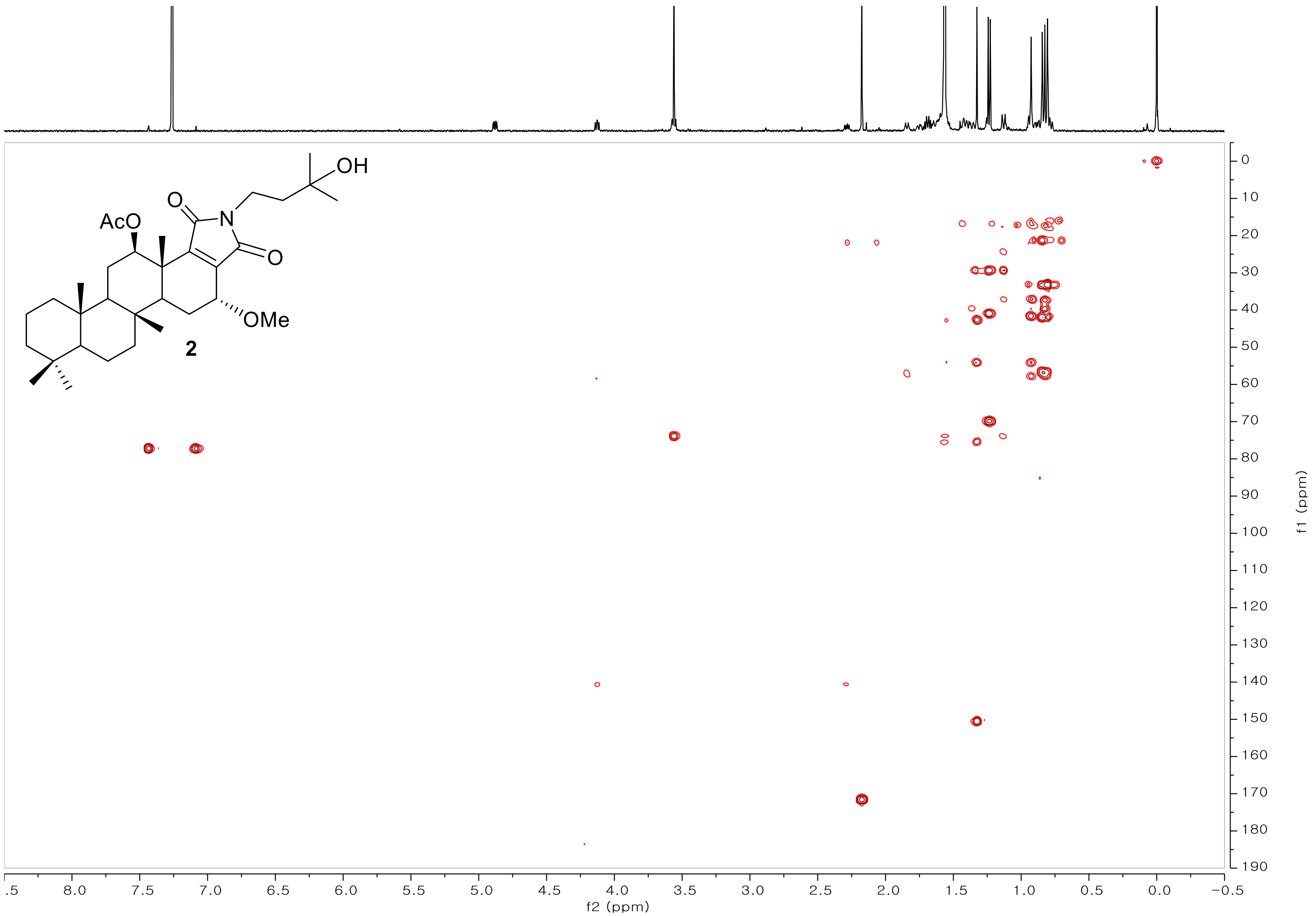

Figure S10. NOESY spectrum of scalalactam B (**2**) in $CDCl_3$ (600 MHz)

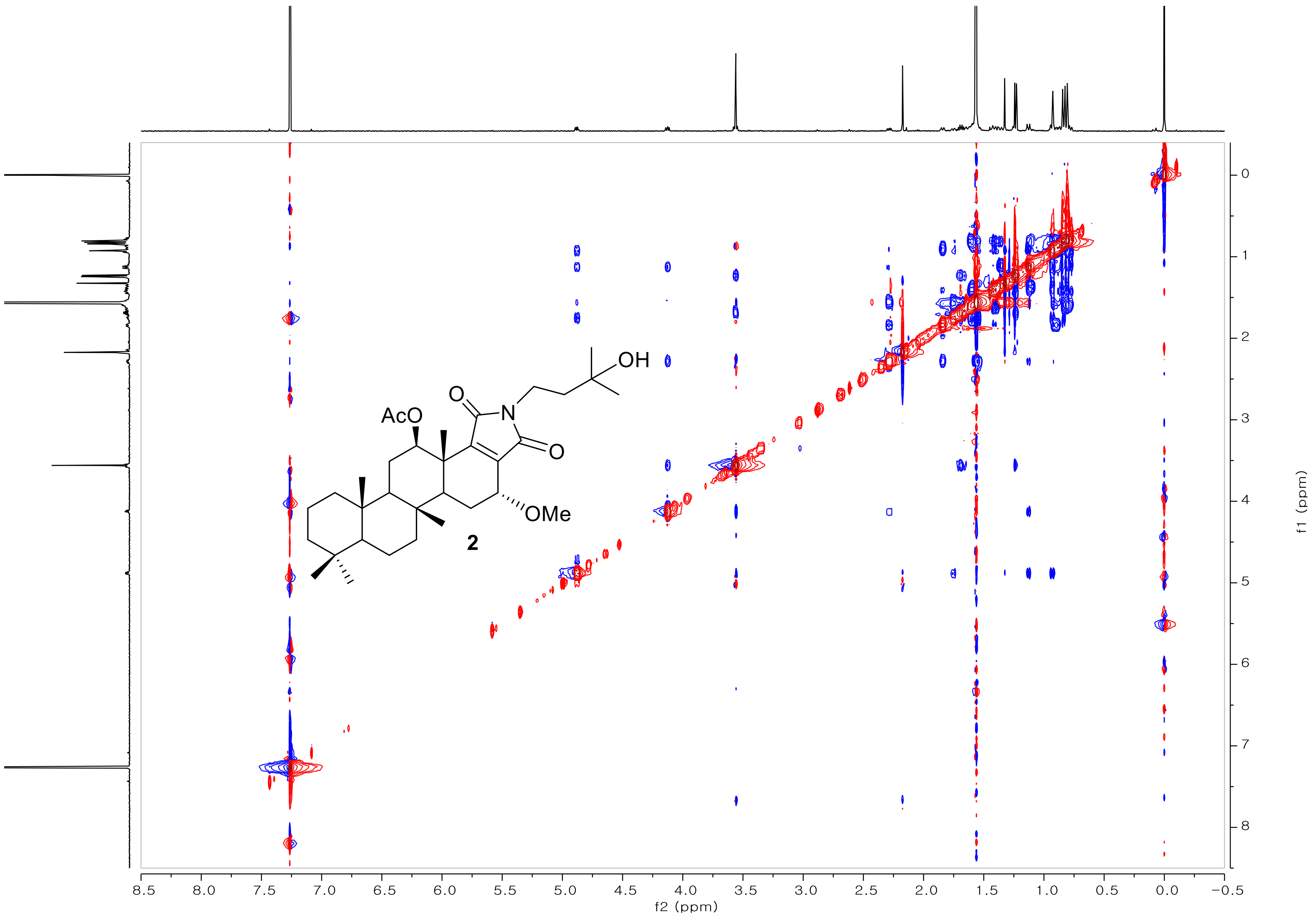

Figure S11. ^{1}H NMR spectrum of scalalactam C (**3**) in $CDCl_3$ (600 MHz)

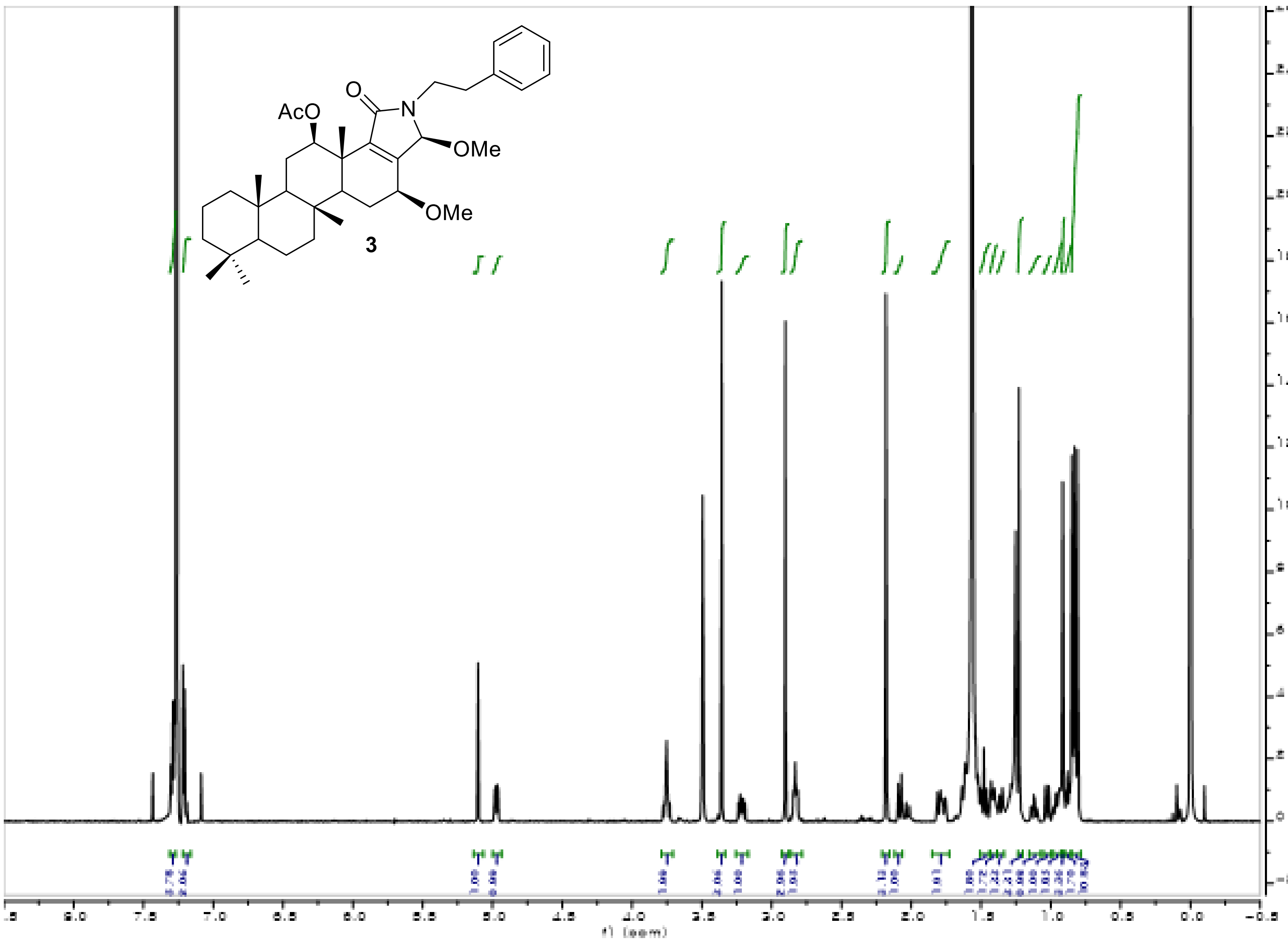

Figure S12. COSY spectrum of scalalactam C (**3**) in $CDCl_3$ (600 MHz)

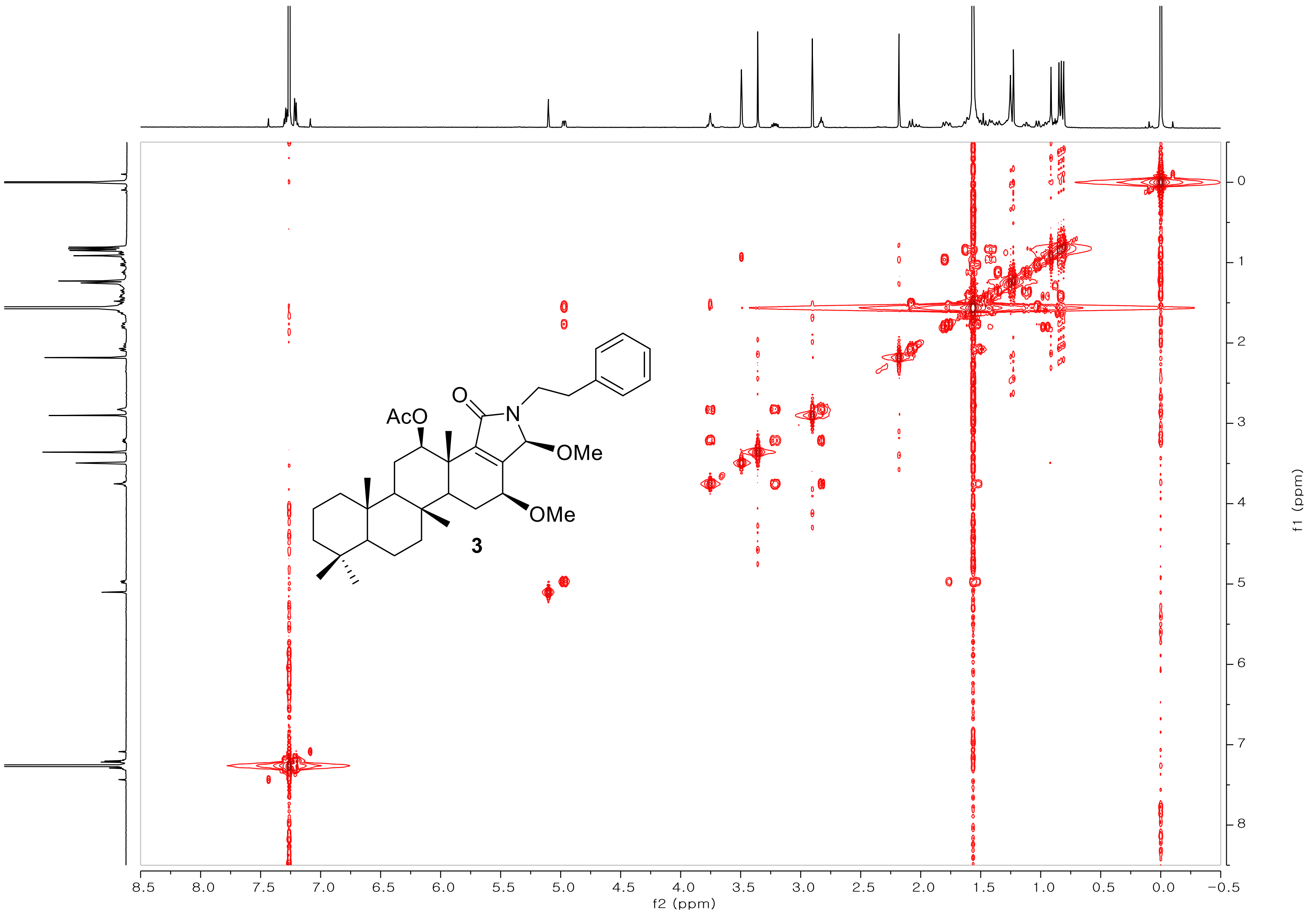

Figure S13. HSQC spectrum of scalalactam C (**3**) in $CDCl_3$ (600 MHz)

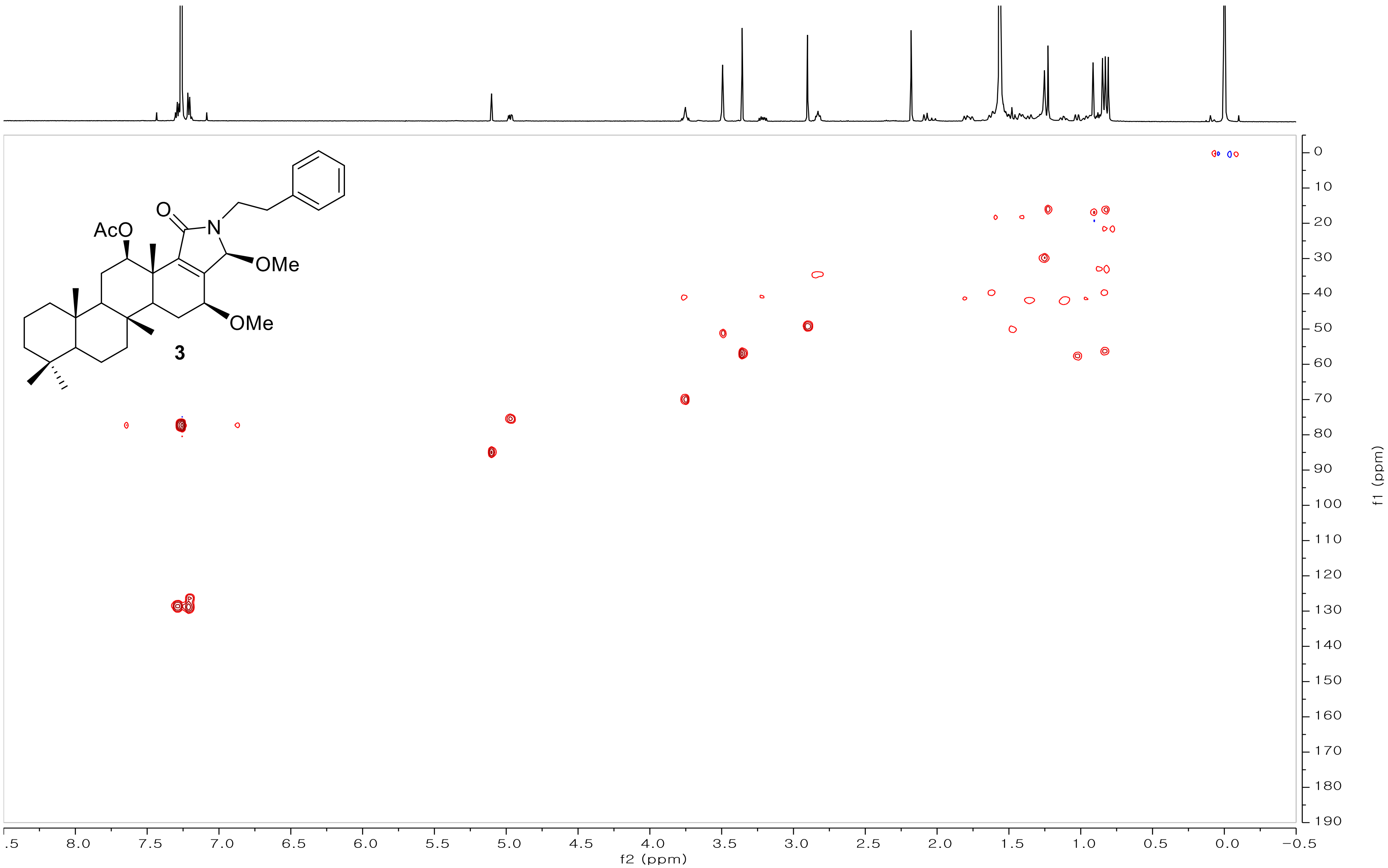

Figure S14. HMBC spectrum of scalalactam C (**3**) in $CDCl_3$ (600 MHz)

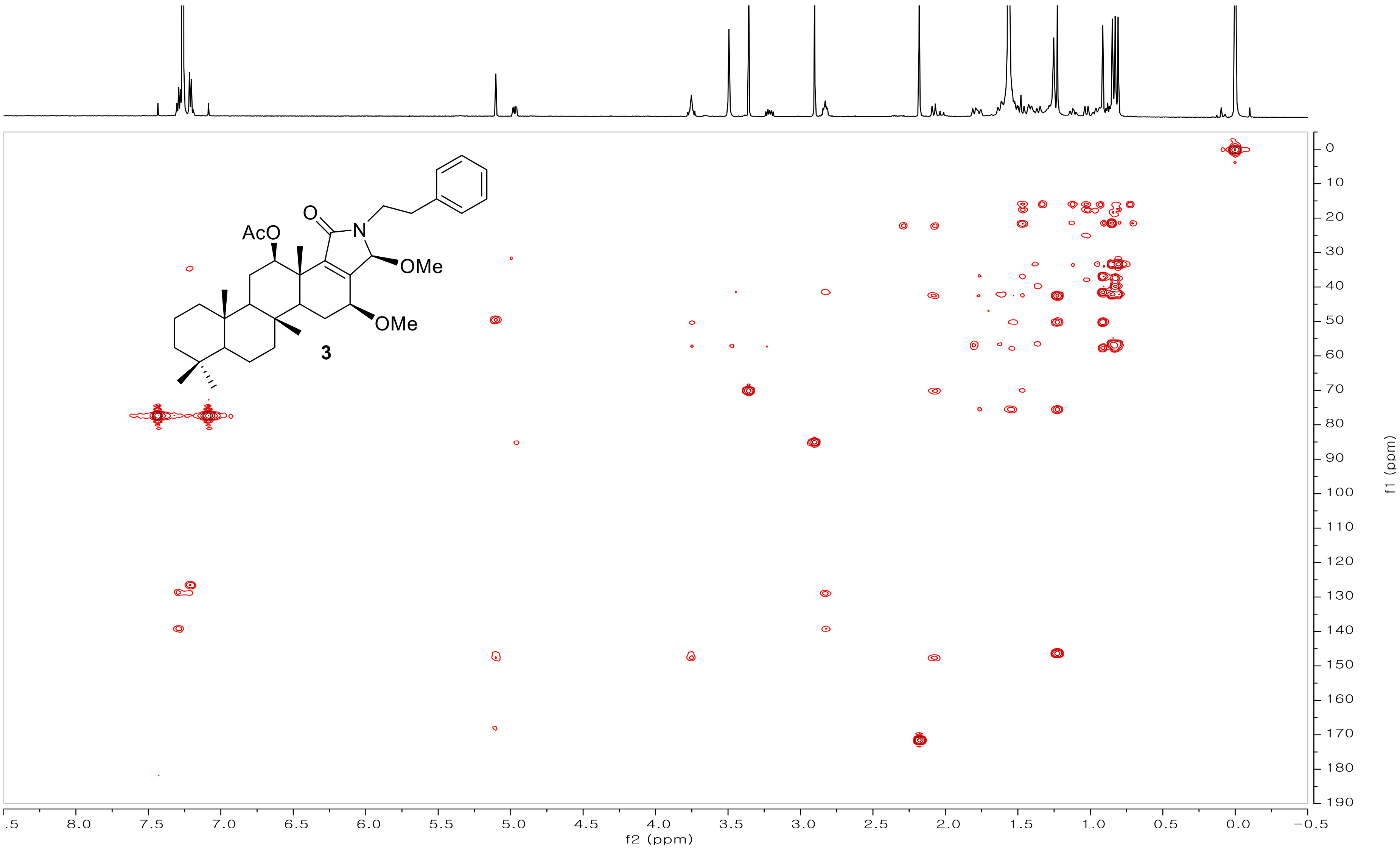

Figure S15. NOESY spectrum of scalalactam C (**3**) in $CDCl_3$ (600 MHz)

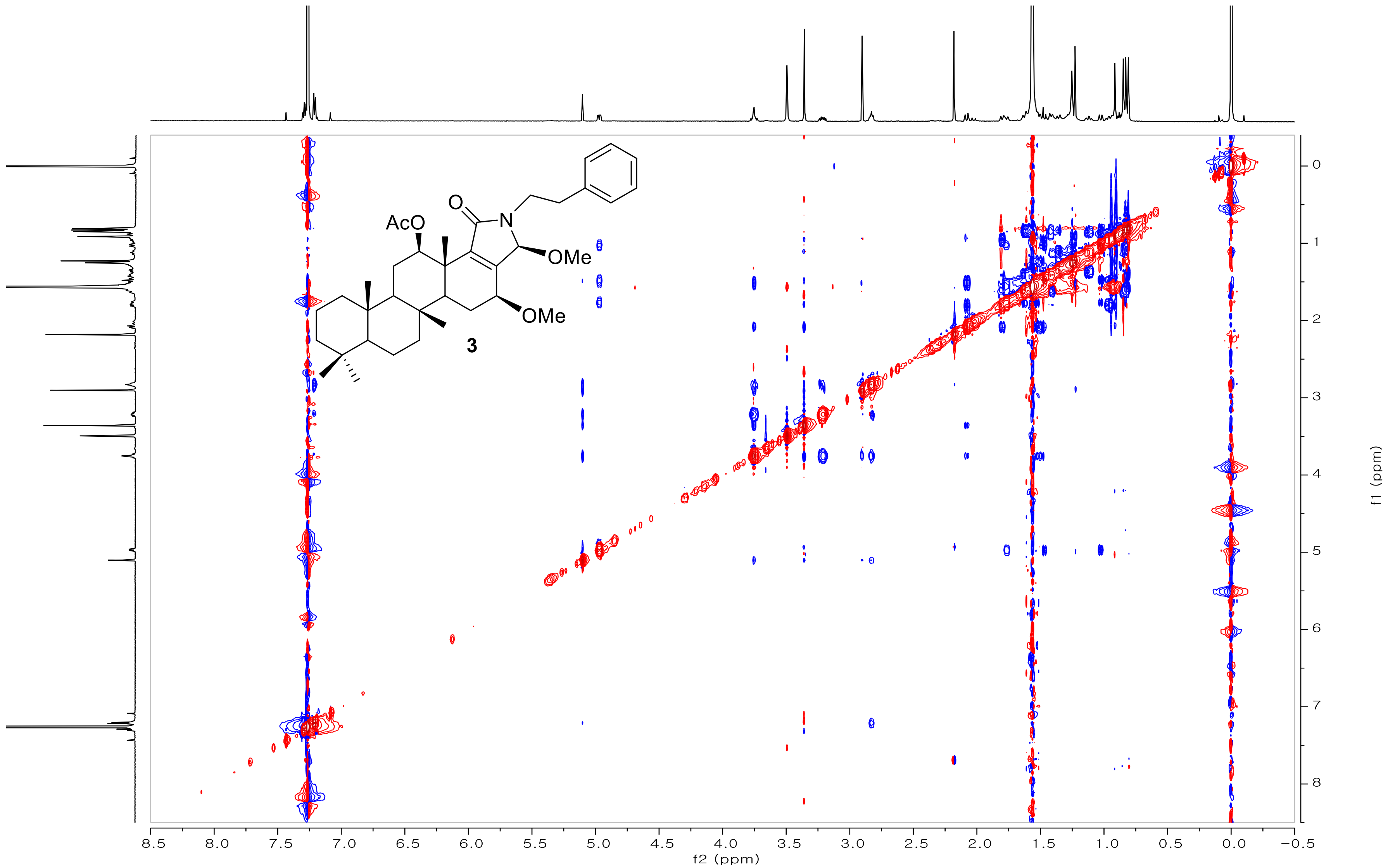

Figure S16. ^{1}H NMR spectrum of scalalactam D (**4**) in $CDCl_3$ (600 MHz)

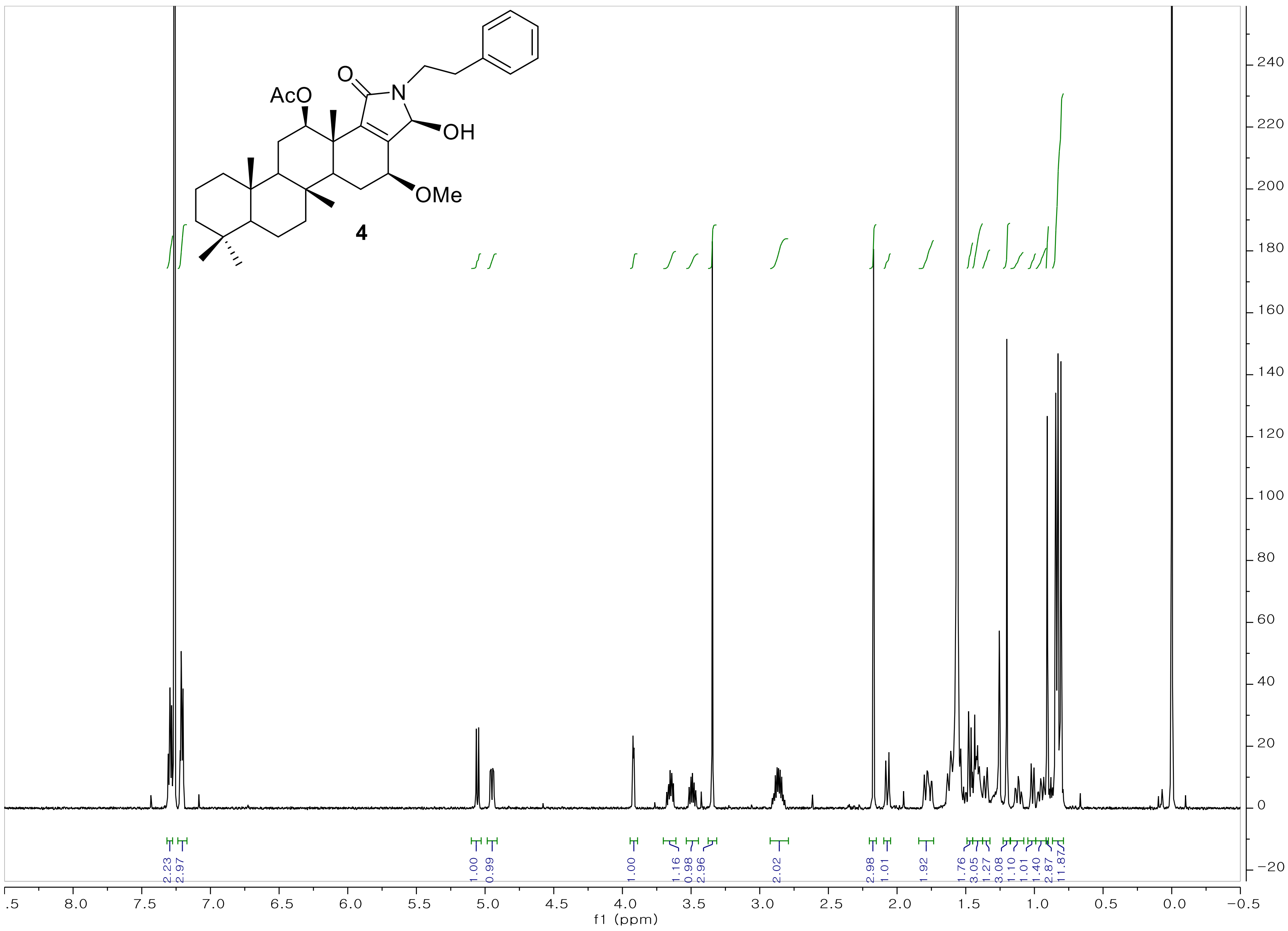

Figure S17. COSY spectrum of scalalactam D (**4**) in $CDCl_3$ (600 MHz)

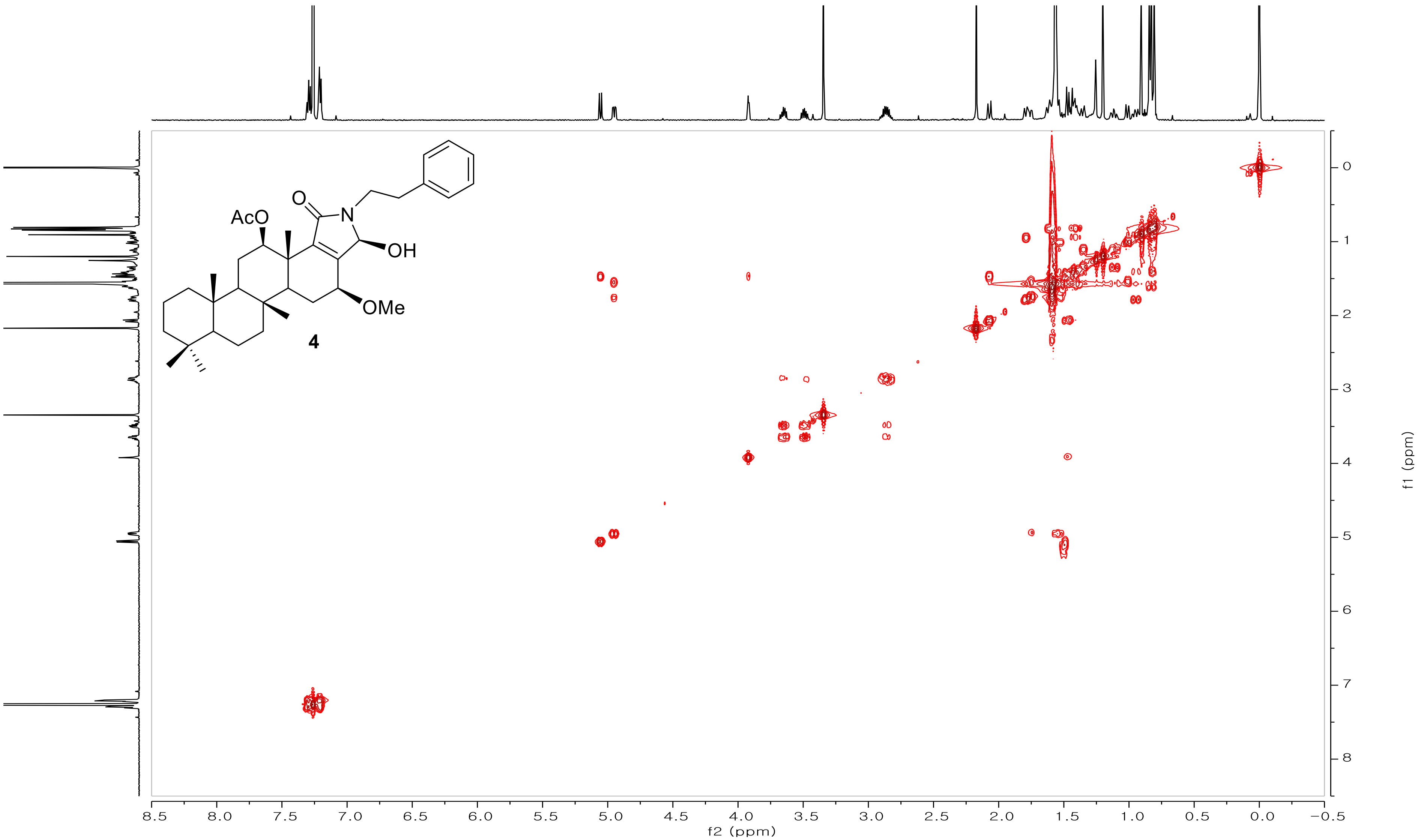

Figure S18. HSQC spectrum of scalalactam D (**4**) in $CDCl_3$ (600 MHz)

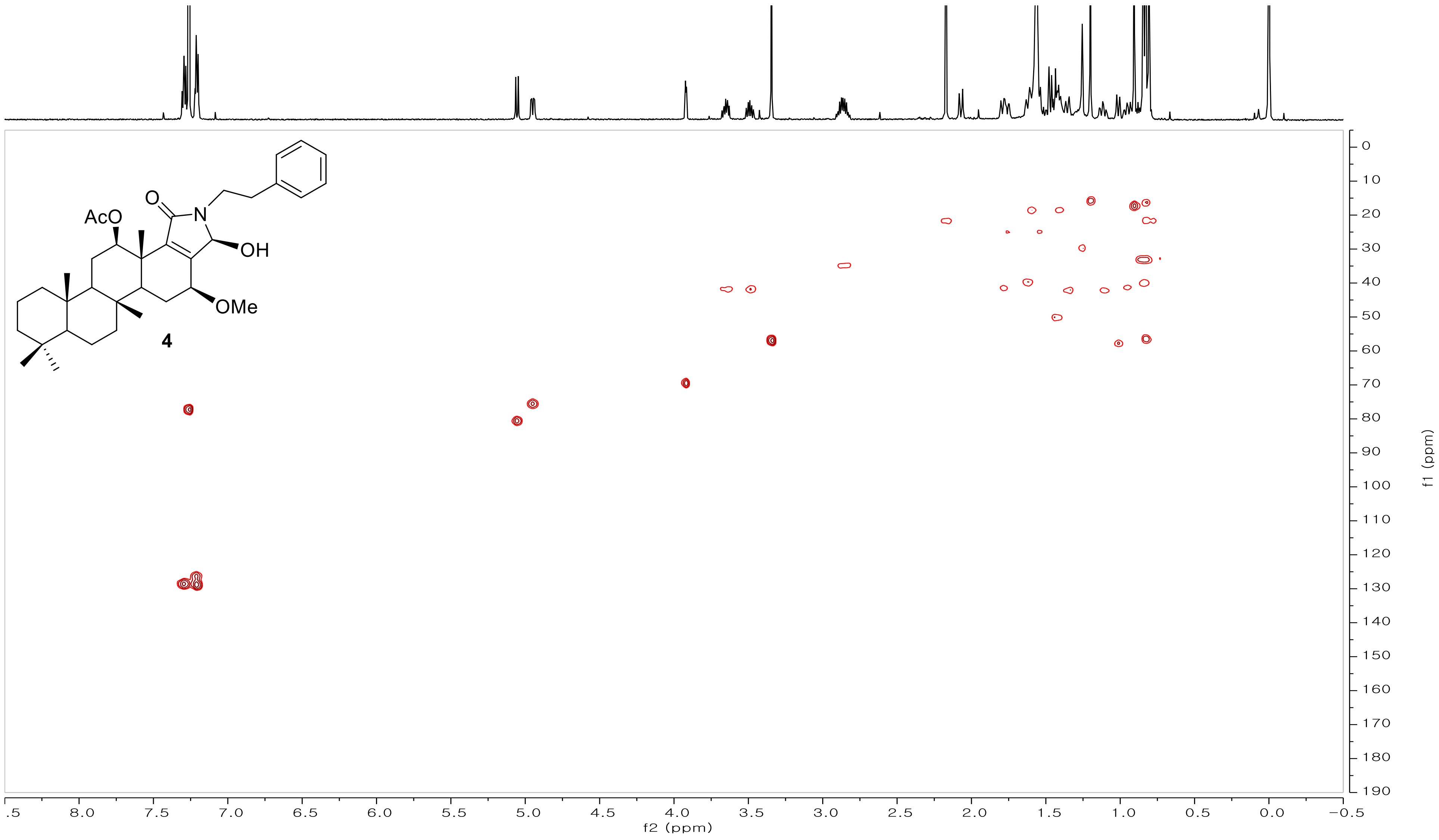

Figure S19. HMBC spectrum of scalalactam D (**4**) in $CDCl_3$ (600 MHz)

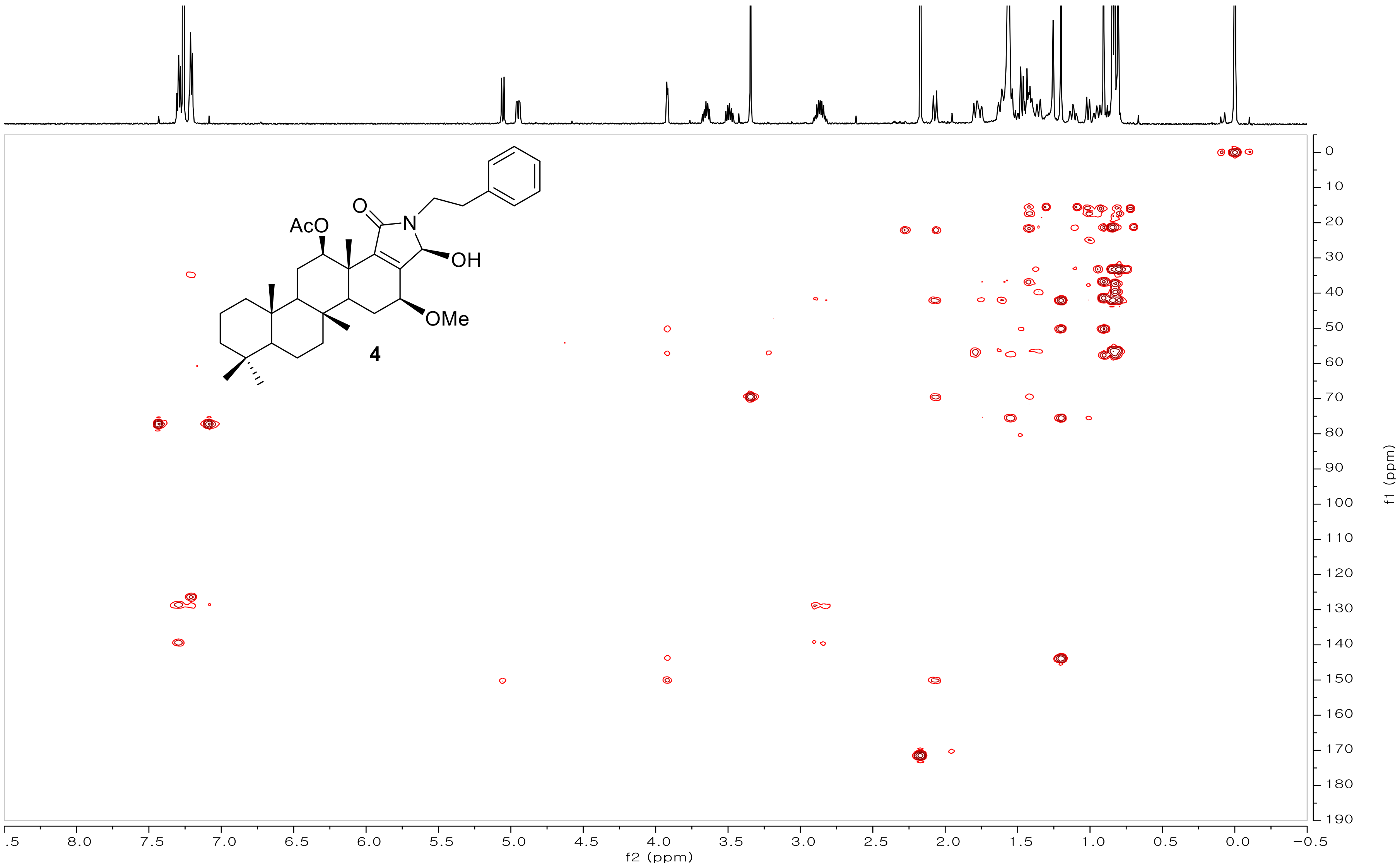

Figure S20. NOESY spectrum of scalalactam D (**4**) in $CDCl_3$ (600 MHz)

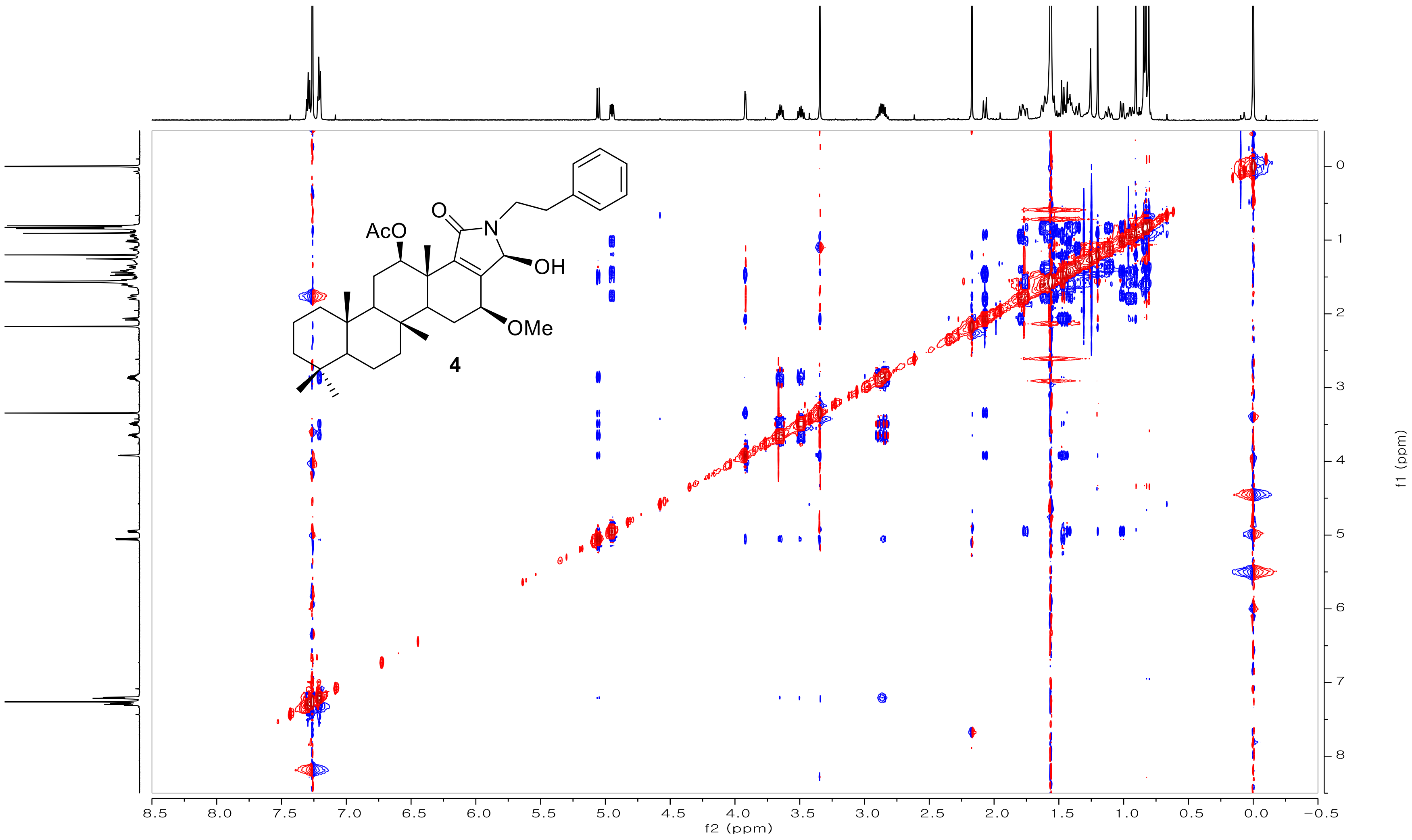

Figure S21. FAB-MS Spectrum of **1**

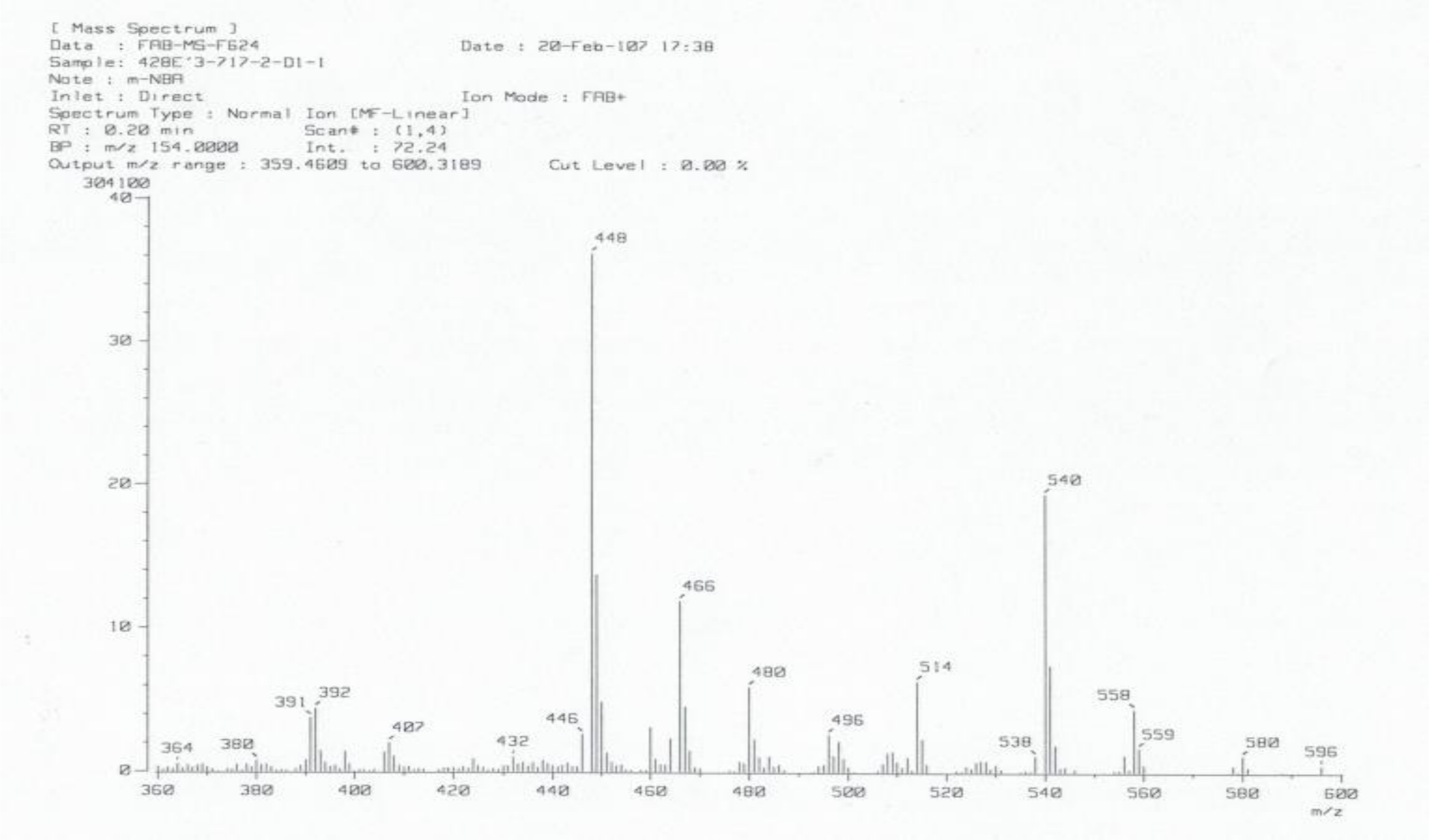

Figure S22. FAB-MS Spectrum of **2**

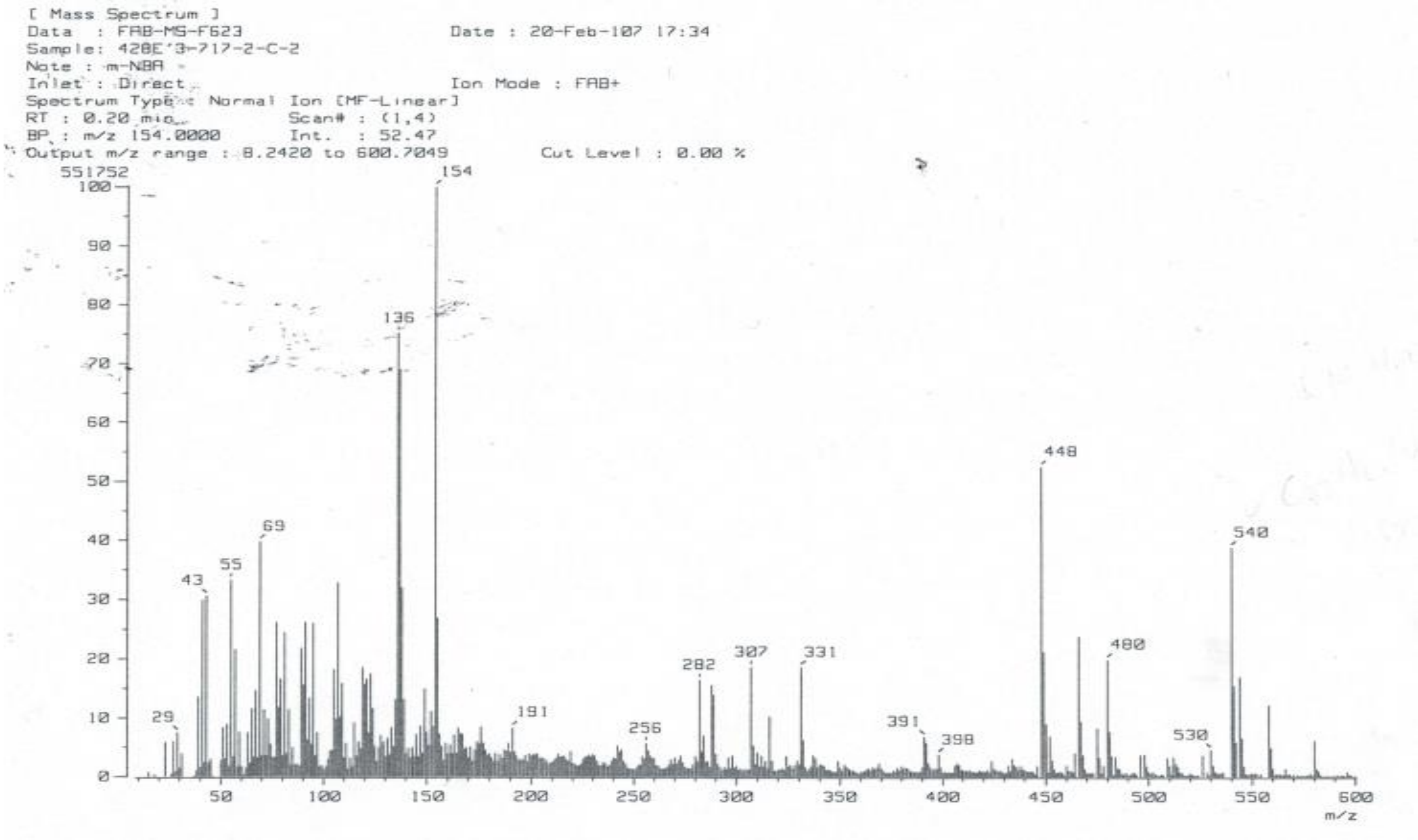

Figure S23. FAB-MS Spectrum of **3**

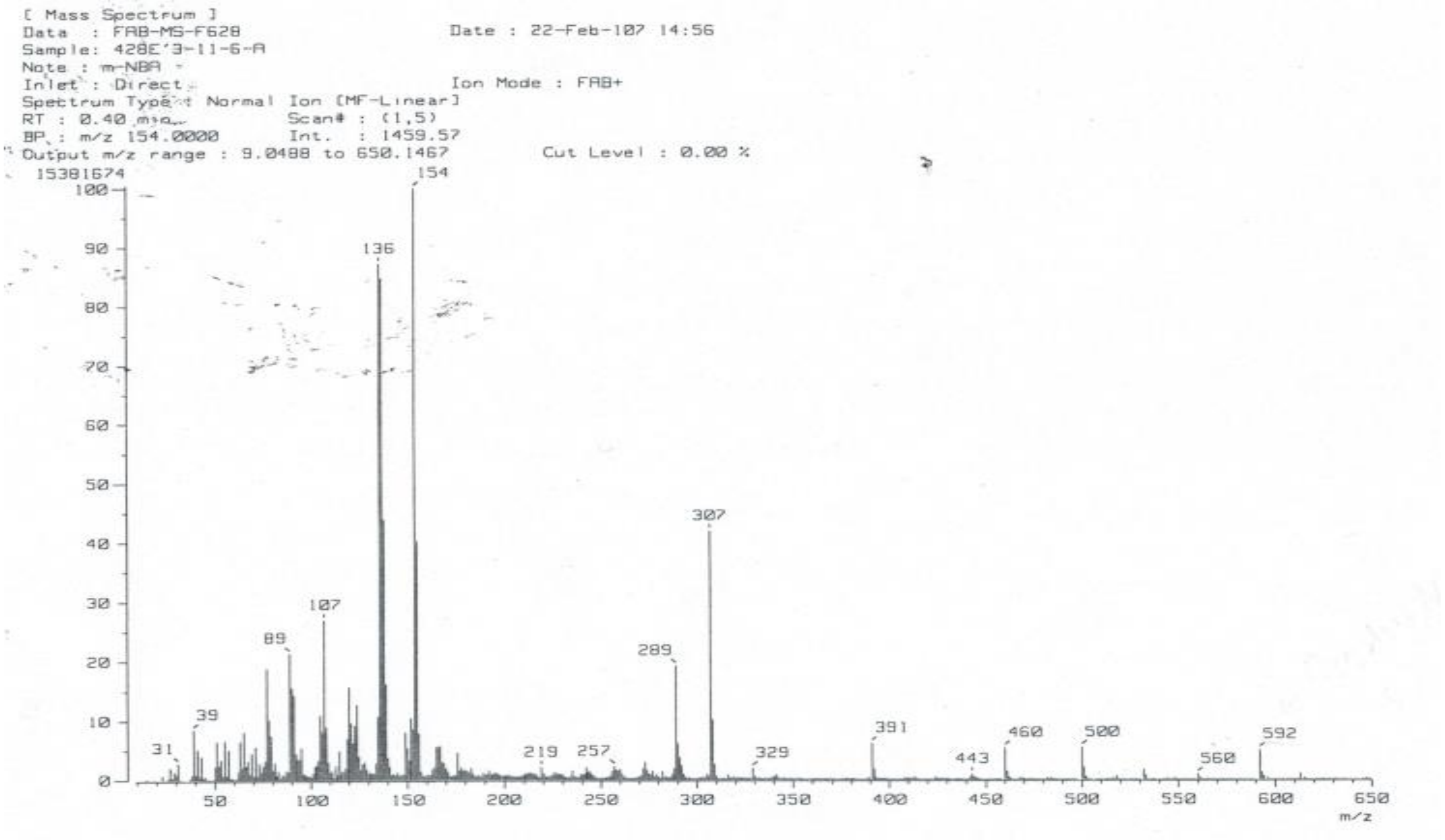

Figure S24. FAB-MS Spectrum of **4**

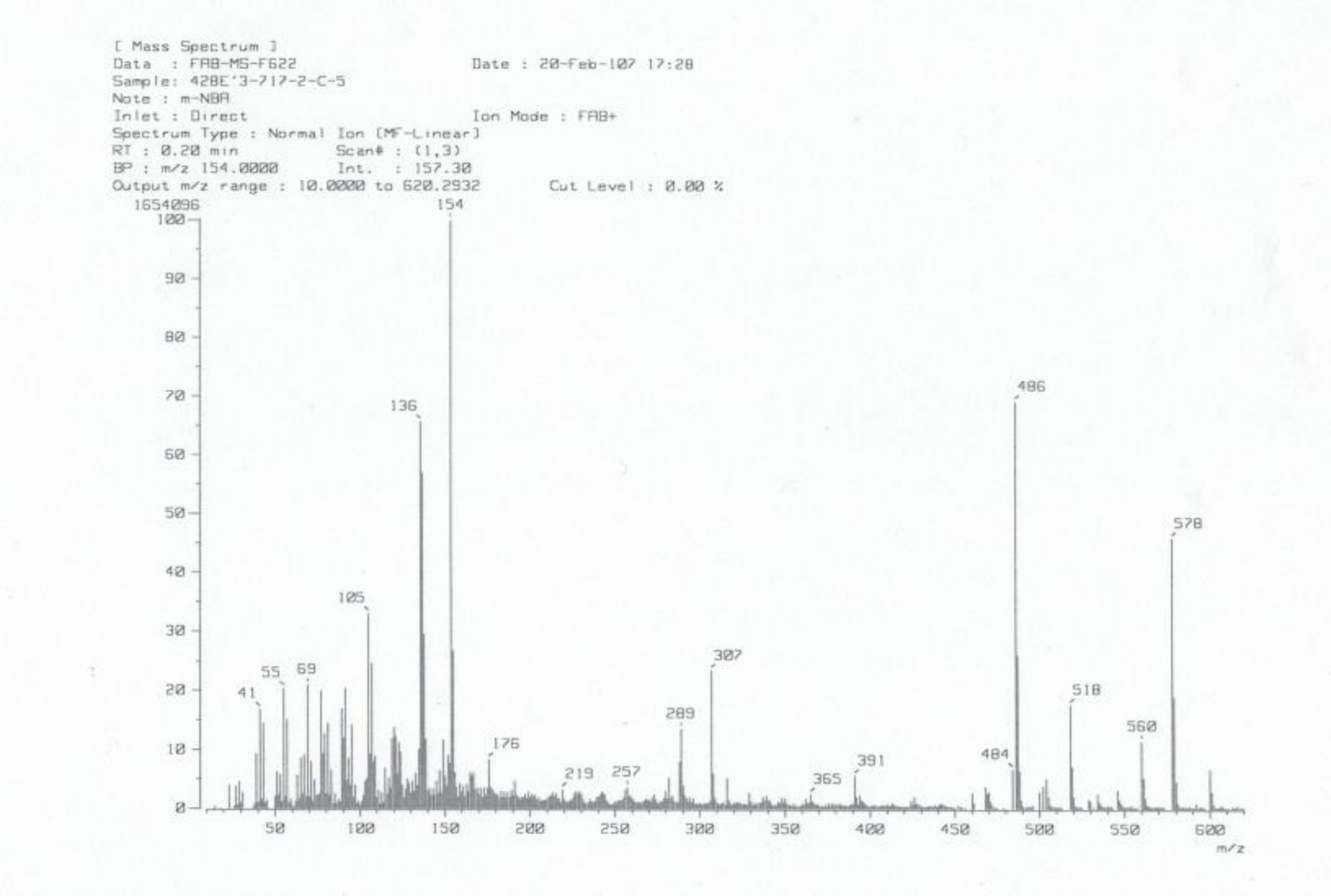

Supplement: Supplementary file 1 [file molecules-23-03187-s001.pdf]
